# Knowledge, attitudes and practices toward skin cancer prevention among Malaysian adults: a cross-sectional online survey

**DOI:** 10.1136/bmjopen-2025-103040

**Published:** 2026-02-22

**Authors:** Ali Haider Mohammed, Bassam Abdul Rasool Hassan, Yen Jun Wong, Loh Hui Ying, Marcus Loh Boon Hong, Annabel Wong Sze Nee, Lo Siew Ying, Dinesh Sangarran Ramachandram, Hawar Sardar Hassan, Lee Jia Jia, Juman Dujaili, Ali Blebil

**Affiliations:** 1 Monash University Malaysia, Bandar Sunway, Malaysia; 2 Department of Pharmacy, Al-Rafidain University College, Baghdad, Iraq; 3 Department of Dentistry, Komar University of Science and Technology, Sulaymaniyah, Iraq; 4 Nuclear Technology Research Centre, Universiti Kebangsaan Malaysia, Bangi, Malaysia; 5 Department of Applied Physics, Universiti Kebangsaan Malaysia, Bangi, Malaysia; 6 Medical School, Swansea University, Swansea, UK

**Keywords:** Knowledge, Attitude, Dermatological tumours, Epidemiology

## Abstract

**Abstract:**

**Objectives:**

To assess the levels of knowledge, attitudes and practices (KAP) toward skin cancer prevention among Malaysian adults and to examine differences in KAP across socio-demographic groups.

**Design:**

Cross-sectional online survey.

**Setting:**

Community-based study conducted in Malaysia using social media recruitment.

**Participants:**

A total of 386 adults aged ≥18 years residing in Malaysia. Most participants were young adults (86.3%), female (55.4%) and of Chinese ethnicity (65.5%). Healthcare professionals were excluded.

**Primary and secondary outcome measures:**

Primary outcomes were levels of knowledge, attitude and preventive practices toward skin cancer, measured using the validated KAP-SC-Q (Knowledge, Attitude and Practice of Skin Cancer Questionnaire) and categorised as poor, moderate or good. Secondary outcomes included differences in KAP across socio-demographic and clinical characteristics, analysed using independent t-tests and χ^2^ tests.

**Results:**

Over half of participants demonstrated poor knowledge of skin cancer (56.0%) and the vast majority showed inadequate preventive practices (84.2%), while attitudes toward skin cancer were predominantly positive (62.4%). Significant differences in mean KAP scores and categorical levels were observed across several socio-demographic variables. Participants with tertiary education had higher knowledge (14.32 vs 12.61) and attitude scores (20.01 vs 15.95; p<0.001) than those with lower education. Individuals with a diagnosis of skin disease had significantly higher knowledge (14.95 vs 13.03; p=0.001), attitude (20.03 vs 18.21; p=0.007) and practice scores (12.10 vs 9.72; p<0.001). Personal history of skin cancer and severe sunburn was associated with better preventive practices but poorer attitudes (p<0.001), and light-skinned participants were more likely to have poor knowledge and attitudes (p<0.05).

**Conclusions:**

Malaysian adults exhibited limited knowledge and very poor preventive practices toward skin cancer despite generally positive attitudes. These findings highlight substantial gaps between awareness and behaviour and support the need for targeted public health interventions to correct misconceptions, improve risk perception especially in high-risk groups and promote effective ultraviolet protection behaviours.

**Strengths and limitations of this study**

- A validated Malaysian version of the KAP-SC-Q (Knowledge, Attitude and Practice of Skin Cancer Questionnaire) was used to measure knowledge, attitudes and practices related to skin cancer.
- A sufficiently large sample size (n=386) was achieved, meeting and exceeding the required minimum for statistical analysis.
- The use of online snowball sampling may have introduced selection bias, particularly toward younger, urban and digitally active participants.
- Self-administered online responses may be influenced by recall bias or social desirability bias.
- The cross-sectional design prevents establishing causal relationships between socio-demographic variables and knowledge, attitudes and practices scores.

## Introduction

 Skin cancer represents a growing global health issue, with over 1.5 million new cases reported in 2020 alone, leading to more than 120 000 fatalities globally.^1^ The projected increase in melanoma cases by 50% from 2020 to 2040 underscores an urgent need for comprehensive studies and interventions, especially as the incidence of skin cancer escalates partly due to heightened exposure to ultraviolet (UV) radiation; a concern exacerbated by the ozone layer’s depletion. This is particularly relevant for equatorial regions like Malaysia, where UV radiation penetrates more intensely due to the ozone layer’s thinning.^2 3^ Local statistics from Malaysia between 2006 and 2014 indicated that basal cell carcinoma was the most common skin cancer type in the central hospital, comprising 34.9% of all skin cancer cases. Additionally, cutaneous lymphoma and squamous cell carcinoma accounted for 25.7% and 20.6% of cases, respectively.^3^

The primary carcinogen for skin cancer, UV radiation (UVR), is well-recognised, yet several other risk factors also play critical roles. Individuals with a familial history of skin cancer are at a significantly elevated risk, with a fourfold increase for squamous cell carcinoma and even higher for basal cell carcinoma and multiple skin cancer types, suggesting genetic predispositions significantly influence skin cancer risks.^4^ Other risk enhancers include the number of moles, existing skin conditions, skin type and tone and the presence of freckles and blisters, which collectively elevate skin cancer susceptibility.

Despite its increasing prevalence, the knowledge and understanding of skin cancer among the Malaysian population remain largely unexplored. Limited research has been conducted on the knowledge, attitudes and practices (KAP) concerning skin cancer in Malaysia, highlighting a gap in public health information and knowledge.^5^ This study aims to bridge this gap by assessing the KAP related to skin cancer among Malaysians, offering valuable insights into critical areas for health communication enhancement.

Given the escalating global and local burden of skin cancer, and the multifaceted risk factors contributing to its prevalence, this study stands as a crucial step towards understanding and mitigating the impact of skin cancer in Malaysia. Therefore, the objective of this study was to assess the levels of KAP toward skin cancer prevention among Malaysian adults and to examine how these levels vary across socio-demographic groups.

## Methods

### Study design and study population

This study employed a cross-sectional design to evaluate KAP concerning skin cancer among Malaysian adults. Conducted online from January to April 2023, this investigation received prior approval from the Human Research Ethics Committee (Project ID:36701). Eligibility criteria required participants to be Malaysian citizens, aged 18 years or older, who consented to participate. Healthcare professionals, including dentists, nurses, pharmacists, podiatrists and veterinarians, were excluded to avoid professional bias. Participants were not engaged in designing, conducting, reporting or disseminating our research.

Given the exploratory nature of this study within the Malaysian context, where similar prior research is lacking, the sample size was calculated assuming a 50% response distribution to the main questions. Using the Raosoft sample size calculator with a 5% margin of error, a 95% CI and an expected 50% response rate, the required sample size was determined to be 385 individuals.

### Study protocol

The approved ethics submission served as the study protocol and guided all methodological processes.

### Survey instrument

A validated and reliable questionnaire was developed by the authors prior to carry out the current study as the first aim of this project was to develop and validate the questionnaire aiming to develop an instrument tool to assess the level of KAP of general public towards skin cancer,^5^ then we did carry out the present study. The developed questionnaire is called KAP-SC-Q (Knowledge, Attitude and Practice of Skin Cancer Questionnaire). The definitive version of the KAP-SC-Q had 108 items, divided into 17 social demographics, 30 knowledge, 32 attitude and 29 practice items.^5^ Knowledge items had an acceptable range of 0.4–2.0 in the Item Response Theory (IRT) analysis, a modern psychometric approach that evaluates the relationship between individual item characteristics (eg, difficulty and discrimination parameters) and respondents’ latent trait levels. The Exploratory Factor Analysis (EFA) revealed that the attitude and practice sections contributed to 34.25% and 52.94% of the total observed variance, respectively. The questionnaire was adapted from a previously validated KAP instrument with strong internal consistency (Cronbach’s α≥0.81 across all domains).^5^ The full questionnaire used in this study is provided in online supplemental file 1, and a summary of its psychometric properties is available in online supplemental file 2.

### Data collection

Participants were recruited using the snowball sampling technique. The poster of the study invitation with its details and survey link was disseminated through social media platforms such as WhatsApp, Facebook, Instagram and LinkedIn. Individuals who were interested in participating in the study would need to click on the survey link, which would direct them to the online questionnaire. The online questionnaire was hosted on SurveyMonkey (SurveyMonkey, San Mateo, California, USA).^6^ The study invitation was reposted weekly to encourage participation and response rate. All participants were required to provide informed consent prior to their participation in this study.

### Patient and public involvement

Patients or members of the public were not involved in the design, conduct, reporting or dissemination plans of this research. The survey was administered to the general population without direct patient recruitment. There are currently no plans to disseminate the results directly to participants.

### Data analysis

A scoring method was used to quantify the levels of KAP toward skin cancer among Malaysian adults. Consistent with the validated scoring approach reported in previous work,^5^ one point was assigned for each correct or positive response and zero points were assigned for incorrect, unsure or negative responses.

The total scores for knowledge (30 items), attitudes (32 items) and practices (29 items) were computed and subsequently categorised into three levels (poor, moderate and good) based on established cut-off points validated during questionnaire development. For example, knowledge scores were classified as poor (0–15), moderate (16–23) and good (24–30). Attitude and practice items were initially measured using a 5-point Likert scale and subsequently dichotomised for scoring, whereby ‘agree’ and ‘strongly agree’ were coded as positive (1), and all other responses were coded as non-positive (0).

Descriptive statistics, including means, SD and frequencies, were used to summarise participant characteristics and KAP levels. Group differences in mean KAP scores were examined using independent t-tests, while associations between socio-demographic variables and categorised KAP outcomes were assessed using Pearson’s χ^2^ tests.

To further examine factors associated with poor skin cancer preventive practices, a multivariable binary logistic regression analysis was conducted. Preventive practice was dichotomised as poor versus moderate/good and entered as the dependent variable. Independent variables were selected a priori based on clinical relevance and previous literature and included age group, sex, education level, place of residence, skin tone, personal history of skin cancer and history of severe sunburn. Adjusted ORs with 95% CIs were reported to quantify the strength of associations.

Exact p values are presented throughout. Statistical significance was set at p<0.05. All analyses were performed using SPSS software (V.26.0; IBM, Armonk, New York, USA).

## Results

### Socio-demographic characteristics of participants

A total of 386 participants completed the questionnaire. The average age of the participants was 27.84±16.9 years old, and the majority were female (55.44%). The most common ethnicity was Chinese (65.54%) with the majority having a tertiary level of educational qualification (76.94%). Most of the participants lived in urban areas (86.53%).

In terms of sun exposure, most of the participants were exposed to the sun for less than 1 hour per day (49.22%). About one-quarter of the participants had a personal history of skin cancer (23.83%), and about one-fifth had a family history of skin cancer (17.10%). About half of the participants were diagnosed with skin disease (46.37%).

Most of the participants had a tan skin tone (49.22%) with type 3 skin type (23.32%) and having a low number of moles (0–50) (82.64%). Almost one-quarter of the participants, however, had unusual moles (23.58%). Half of the participants did not experience any hair loss or hair thinning problems (50.52%), and about two-thirds of the participants did not have freckles (61.14%). In addition, approaching one-third of the participants had a severe sunburn that resulted in blisters (30.57%). Further details were shown in online supplemental table 1.

### Level of knowledge, attitude and practice toward skin cancer

**Figure 1:**
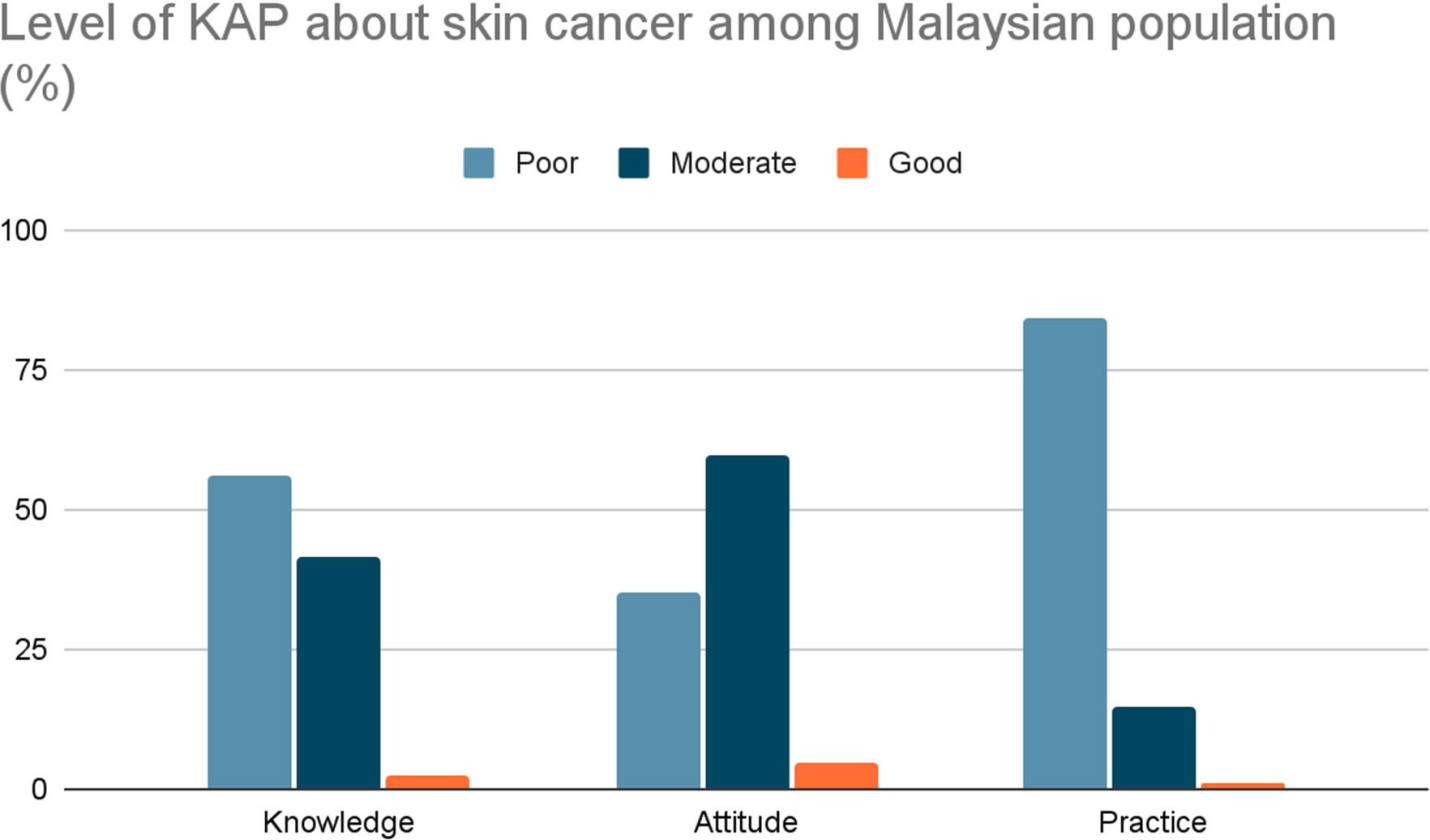
The level of knowledge, attitude and practice towards skin cancer among participants. KAP, knowledge, attitude and practice.

From the given data, it was noted that the participants had a suboptimal level of knowledge about skin cancer (56.0%) with 84.2% showed inadequate preventive measures. This underscores a significant deficiency in the understanding of the disease, which could be a factor contributing to the lack of awareness in practice to help prevent skin cancer. Interestingly, most participants had a positive attitude towards skin cancer, which showed a good sign in promoting the awareness in terms of knowledge and practice towards skin cancer, as demonstrated in figure 1.

Although the overall mean attitude score appeared moderate, individual attitude items showed mostly positive responses (agree or strongly agree). These favourable perceptions did not translate into preventive behaviour, resulting in poor practice scores.

### Participants’ knowledge of skin cancer

Overall, it was noted that knowledge about skin cancer among the Malaysian participants could be further improved (online supplemental file 3). Encouragingly, there were 79.79% of participants correctly identified that individuals with a family and/or personal history of skin cancer are more susceptible to skin cancer. The understanding of skin cancer risk factors is crucial for early detection and prevention of the disease. However, it is concerning that only 39.90% of the participants recognised that UVR from the sun is not the sole cause of skin cancer, as there are other risk factors involved.

Additionally, a substantial number of participants (70.73%) correctly acknowledged that skin cancer screening involves the identification of moles, birthmarks or other marks that are unusual in colour, size, shape or texture, where they could also routinely self-examine their skin condition.

There were, however, misconceptions regarding skin cancer among the participants. For instance, 18.65% of them thought that people with skin that burns or freckles easily are at a lower risk of getting skin cancer, while 42.23% were unsure about this aspect. This misconception might lead to inadequate sun protection measures for those with such skin characteristics. Similarly, about one-quarter of the participants (26.68%) thought that sunscreen with Sun Protection Factor (SPF) 30 refers to the level of protection a sunscreen provides against ultraviolet B (UVB) radiation; theoretically, SPF 30 means it would take approximately 30 times longer for erythema (sunburn) to occur on protected skin compared to unprotected skin, however, 22.28% were unsure about this, possibly indicating a lack of awareness about proper sunscreen use.

Furthermore, about two-thirds of the participants (68.39%) considered supplements containing antioxidants as a protective strategy to reduce the risk of skin cancer. While antioxidants are beneficial for overall health, their specific role in preventing skin cancer remains uncertain, suggesting a need for more accurate information dissemination.

### Participants’ attitude towards skin cancer

In general, participants showed an encouraging attitude towards skin cancer; nevertheless, there were several misconceptions and uncertainties which should be addressed (online supplemental file 3). On a positive note, more than half of the participants (60.62%) agreed that skin cancer is a skin problem that can cause death, highlighting their recognition of the severity of the disease and the importance of early detection and prevention. A similar proportion of participants (57.77%) agreed that skin cancer is highly preventable, demonstrating good awareness of its preventability and the potential impact of educational efforts in reducing its incidence.

However, almost half of the participants (45.60%) disagreed that skin cancer is totally incurable, indicating there might be complacency towards preventive measures and early intervention in treating skin cancer. In addition, a similar number of participants (44.04%) agreed that the number of moles does not affect the risk of getting skin cancer, potentially leading to a lack of attention to changes in moles that could delay the detection of skin cancer. In addition, only 33.68% of them agreed that melanoma skin cancer risk is doubled in people with freckles, suggesting a need for intervention in educating the public regarding the relationship between freckles and skin cancer risk.

Amidst these misconceptions, participants expressed a positive attitude towards skin cancer prevention. For instance, there were 57.51% of participants who agreed that individuals with dark skin require the same protection and regular examination as those with lighter skin types, indicating a recognition of the importance of sun protection for all skin types. Additionally, 58.03% agreed that observing any abnormalities, rashes or inflammation on the skin is a screening method for skin cancer, showcasing their awareness and vigilance in identifying potential signs of the disease.

### Participants’ practice towards skin cancer

The findings showed that participants would require more support in guiding them to improve their practice towards skin cancer prevention (online supplemental file 3). While a significant number of participants performed essential preventive approaches, there were areas which required further attention and improvement. For example, in terms of self-examination, more than half of the participants (60.43%) sometimes, often or always conduct thorough annual skin self-examinations. However, almost half of them (48.70%) did not visit any healthcare professionals for skin examination on an annual basis.

Approximately 20.0–40.0% of the participants did not practice adequate preventive measures when being exposed to the sun, where they never wore any hat with a wide brim to shade their face, head, ears and neck or used UV-protective clothing when going outside, rarely or never reapply sunscreen after excessive swimming or sweating. For activities involving prolonged sun exposure, for example, swimming, almost half of them (44.15%) did not reapply sunscreen after towelling off. Additionally, only 33.01% of the participants used a stronger ultraviolet A (UVA) and ultraviolet B (UVB) radiation, where UVA penetrates deeper into the skin and contributes to photoaging and carcinogenesis, while UVB is primarily responsible for sunburn and direct DNA damage, with SPF50 or higher, suggesting a need for educational intervention on the importance of using adequate sun protection.

In certain occupational settings which might be subject to a higher risk of skin cancer, about one-third of the participants (32.64%) did not undergo skin biopsy tests according to the recommended screening schedules.

### Differences between socio-demographic variables and KAP score mean (Predictors to KAP categories?)

Based on online supplemental table 2, several key insights emerge regarding the KAP related to skin cancer among different socio-demographic groups among the participants. Those with a personal or family history of skin cancer, despite having lower attitude scores (16.89 and 16.85, both with p<0.001), demonstrate better practices (13.14 and 13.65, with p<0.001), suggesting more preventive behaviours in these high-risk groups. Individuals with tertiary education have superior knowledge (14.32, p=0.032) and attitudes (20.01, p<0.001) towards skin cancer, which implies the importance of education in promoting awareness. However, this does not translate to better practice scores (10.63, p=0.161), suggesting a gap between awareness and actual preventive behaviours.

Moreover, people diagnosed with skin diseases show higher KAP scores (knowledge: 14.95, p=0.001; attitude: 20.03, p=0.007; practice: 12.10, p<0.001), underlining the crucial role of existing skin conditions in shaping skin cancer awareness and prevention. Additionally, individuals who have experienced severe sunburns that cause blisters display poorer attitudes towards skin cancer (17.48, p=0.002) but exhibit better practices (12.33, p<0.001), indicating a possible dichotomy between attitudes and practices in those with direct sun damage. Lastly, skin tone and type significantly influence attitudes and practices, with individuals having light skin tones showing poorer attitudes (13.73, p=0.006) and those with skin type 1 (burns easily, never tans, sensitive to UV exposure) having inferior practices (9.94, p=0.007). This could be due to higher vulnerability to sunburns and skin cancer, underscoring the need for more focused awareness programmes for these demographics as shown in online supplemental table 2.

### Significant association between the level of KAP and demographic variables

The data in online supplemental table 3 provides a significant association between skin cancer knowledge and personal history of skin cancer, family history of skin cancer, diagnosis of other skin diseases and skin tone. For instance, 67.4% of individuals with a personal history of skin cancer have poor knowledge about it (p=0.040). This is surprising as we would expect these individuals to be more informed about the disease. Similarly, those with a family history of skin cancer show 60.6% with poor knowledge, which is slightly lower but still significant. On the other hand, 48.0% of individuals diagnosed with other skin diseases have poor knowledge about skin cancer (p=0.014), implying a diagnosis of any skin condition might increase awareness about skin diseases. Finally, an important finding is that 93.3% of light-skinned individuals, who are more susceptible to skin cancer, have poor knowledge about the disease (p=0.036).

Interestingly, individuals with a personal (52.7%, p<0.001) or familial (54.5%, p<0.001) history of skin cancer—groups typically expected to be more vigilant—also display poor attitudes. The data reveals an alarming trend: those most susceptible to skin cancer, like light-skinned individuals (73.3%, p=0.033) and those with severe sunburn history (48.3%, p=0.001), often maintain poor attitudes towards the disease as shown in online supplemental table 4.

Moreover, online supplemental table 5 reveals a significant association between skin protection practices and a person’s medical history related to skin cancer and skin diseases. Notably, individuals with a personal history of skin cancer had a higher percentage of moderate skin protection practices (32.6%) compared with those without any history (9.2%). A similar pattern is observed for individuals with a family history of skin cancer, with 34.8% exhibiting moderate skin protection practices as opposed to 10.6% in those without a family history. These findings underscore the influence of personal and family medical history on the implementation of skin protection practices.

Lastly, online supplemental table 6 presents the results of the multivariable logistic regression analysis examining factors associated with poor skin cancer preventive practices among Malaysian adults. After adjustment for relevant socio-demographic and clinical variables, male sex was significantly associated with higher odds of poor preventive practices (adjusted OR=1.85, 95% CI 1.22 to 2.80, p=0.004). Participants with non-tertiary education also had significantly increased odds of poor preventive practices compared with those with tertiary education (adjusted OR=2.14, 95% CI 1.39 to 3.30, p=0.001). Light skin tone was independently associated with higher odds of poor preventive practices (adjusted OR=1.76, 95% CI 1.05 to 2.94, p=0.032), as was a history of severe sunburn (adjusted OR=1.67, 95% CI 1.10 to 2.54, p=0.016). In contrast, participants with a personal history of skin cancer were less likely to report poor preventive practices (adjusted OR=0.58, 95% CI 0.36 to 0.93, p=0.024). Age ≥30 years and urban residence were not significantly associated with poor preventive practices in the adjusted model.

## Discussion

### Knowledge, attitude and practice toward skin cancer

Skin cancer has been recognised as a growing concern globally and particularly in Malaysia.^7^ However, limited studies in Malaysia have assessed the public’s KAP concerning skin cancer. This study seeks to fill this literature gap by identifying the Malaysian’s KAP on skin cancer using adapted and validated questionnaire.

The findings revealed a significant knowledge gap regarding skin cancer. Many participants were unaware that UVR from the sun is a leading cause of skin cancer, consistent with the findings by Schadendorf and colleagues.^8^ Moreover, most participants did not realise that men and those with lighter skin or frequent burning/freckling are more susceptible to skin cancer. Another common misconception is that those with darker skin were less susceptible.^9^ Moreover, the misbelief that melanoma only develops in sun-exposed areas is alarming, considering its deadly nature.^10^ Despite these gaps, most participants recognised the importance of early detection and intervention.

In terms of attitude, while most of the participants understood the seriousness of skin cancer, several misconceptions might hinder effective prevention. A lack of regular skin examinations and misconceived benefits of sunbathing further demonstrated the need for targeted public health campaigns in addressing the misinformation regarding skin cancer and protective approaches.^11 12^

Regarding practice, most participants were unfamiliar with the importance of self-examination for early detection of skin cancer. Although many of the participants had great awareness of sunscreen use with its benefits, the application remained inconsistent, especially among men.^13 14^ Recommendations for sun-protective clothing were also limited, with most unaware of the best fabric choices for UV protection.^15^

Our results align with research from other regions showing a consistent gap between awareness and preventive behaviours. Among Jordanian medical students, high knowledge of skin cancer risk factors did not translate into action, with 61.5% reporting they had never performed a skin self-examination and 20% not using sunscreen at all.^16^ Likewise, a multicentre study in Saudi Arabia found that although students recognised the importance of UV protection, 30% never used sunscreen and 57% failed to reapply it, with usage driven mainly by cosmetic reasons rather than cancer prevention.^17^ These findings reinforce that knowledge and attitudes alone do not reliably lead to protective behaviours, emphasising the need for targeted interventions that improve practical prevention habits.^16^,^20^

Similar patterns have been reported across Southeast Asia. In Thailand, a survey of more than 10 000 secondary school students in Bangkok found that sun protection behaviours were poor compared with Western populations, with particularly low use of sunscreen, hats and protective clothing and consistently poorer protection among male students.^18^ In Indonesia, university students showed gaps between awareness of UVR and actual photoprotective behaviour, with many reporting inconsistent use of sunscreen and other protective measures despite reasonable knowledge levels.^19^ In Singapore, a nationwide survey of adults identified low overall sun protection scores and showed that younger age, male sex, darker skin type and lower education were associated with longer sun exposure and poorer sun safety habits.^20^ These regional findings are consistent with our results and suggest that socio-demographic factors and perceived low vulnerability contribute to suboptimal prevention behaviours across high-UV Asian settings.

Cultural and aesthetic influences may also affect sun protection behaviours in Malaysia. Similar to Middle Eastern and other Asian populations, sunscreen use is often perceived primarily as a cosmetic product rather than a health necessity, particularly among men. For example, a study in Saudi Arabia reported that sunscreen was mainly used to prevent tanning rather than to reduce skin cancer risk, with 38.2% citing cosmetic reasons as their primary motivation.^17 18 20^ Such beliefs may reduce engagement in preventive practices, despite generally favourable attitudes toward skin cancer.

### Differences and associations between socio-demographic and KAP

The study found that those with a history of skin cancer had poor knowledge about the disease, contrary to expectations. A possible reason is health literacy; low literacy levels can impede understanding of skin cancer information. Unreliable sources and poor communication between doctors and patients could also contribute. Men showed negative attitudes towards skin cancer, supported by prior evidence showing gender differences in risk perception and health-seeking behaviour.^21^,^23^ Women, on the other hand, practiced better preventive measures, with studies like^22 24 25^ confirming women’s higher sunscreen usage. This can be due to health literacy, societal expectations or both. It is concerning that men above 65 are more susceptible to melanoma than their female counterparts, as noted by previous epidemiological evidence.^23^

Moreover, higher education correlates with better skin cancer attitudes, aligning with findings from prior population-based studies.^26^ Those with more education understand skin cancer risks and are proactive about prevention. Public health efforts should focus on low-education populations, offering accessible, clear information. Disturbingly, our analysis showed that light-skinned individuals, especially those with a history of severe sunburns, neglect skin cancer precautions. Prior research links sunburn history to sunscreen use patterns and long-term skin cancer risk.^27 28^ Those sunburned before the age of 25 face elevated skin cancer risks. Light-skinned individuals, especially of Chinese descent, are more at risk, as seen in regional studies documenting increased vulnerability among lighter-skinned Asian populations.^29 30^

Lastly, concerning prevention, Malaysians show poor practices, influenced by ethnicity, education and skin type. Many falsely believe that darker skin provides immunity, resulting in inadequate self-checks, as stated by.^31^ Education plays a pivotal role, with studies like these demonstrating the positive influence of education on skin cancer awareness.^21 32^ Finally, skin type significantly affects practices, with studies indicating that fair-skinned individuals face higher risks and therefore require targeted awareness campaigns.^29 33^

Therefore, the study findings stress the need for tailored public health campaigns to address misconceptions and emphasise evidence-based knowledge. Collaborations with dermatological bodies might enhance credibility and trust in the information provided. Public health measures should highlight the importance of early detection and the need for consistent sun protection across all skin types.

These findings support the need for national public health strategies that emphasise practical preventive behaviours rather than solely increasing awareness. Campaigns should prioritise teaching correct sunscreen use, encouraging routine skin self-examination and promoting protective clothing, particularly targeting young adults and men. Collaboration with universities, primary care clinics and social media platforms can broaden outreach. Considering that sunscreen use is often driven by cosmetic rather than health motives in regional populations,^17^ health campaigns may benefit from framing sun protection benefits in ways that appeal to both aesthetic and medical prevention outcomes.

### Article summary

To our best knowledge, this was one of the pioneering studies that explore the KAP towards skin cancer in Malaysia. This allowed us to identify potential areas for improvement through targeted educational intervention in imparting information to the public in an effective manner.

The generalisability of this study is limited by the use of social media-based snowball sampling, which may disproportionately represent younger, urban, more educated and digitally active individuals. The sample was also skewed toward Chinese respondents, which does not fully reflect Malaysia’s multiethnic demographic structure. Although the required sample size was achieved, representativeness cannot be guaranteed due to potential selection bias and the self-selection nature of voluntary online participation.

The data were collected via self-reported responses, which may introduce social desirability bias, particularly for preventive practices such as sunscreen use and skin self-examination. Healthcare professionals were intentionally excluded to avoid inflating public knowledge and preventive behaviour scores; however, this restriction limits comparison between the general population and clinically trained individuals.

Furthermore, the cross-sectional design precludes causal inference, and observed associations should not be interpreted as predictive relationships. Future studies should consider probability-based or stratified sampling methods to enhance representativeness.

## Conclusion

This study concluded that most participants demonstrated suboptimal levels of knowledge and practice concerning skin cancer and its prevention. Encouragingly, they had moderately positive attitudes towards this skin condition. This inconsistency between awareness and actual preventive measures highlights a critical gap that requires urgent attention. Thus, this study serves as a compelling call to action for healthcare professionals, educators and policymakers in Malaysia to recognise and address the complex landscape of skin cancer awareness. The urgent need to bridge the gap between knowledge and practice is imperative, and targeted efforts are essential to make strides in skin cancer prevention and early detection within the Malaysian population. The collective effort will contribute to safeguarding public health and potentially save lives by reducing the incidence and impact of this serious but preventable disease.

## Supplementary material

10.1136/bmjopen-2025-103040 online supplemental file 1

10.1136/bmjopen-2025-103040 online supplemental file 2

10.1136/bmjopen-2025-103040 online supplemental file 3

10.1136/bmjopen-2025-103040 online supplemental file 4

## Data Availability

Data are available in a public, open access repository.
