# Supplementary material for: Knowledge, attitudes and practices toward skin cancer prevention among Malaysian adults: a cross-sectional online survey
Source: BMJ Open. 2026 Feb 22;16(2):e103040. doi: 10.1136/bmjopen-2025-103040 (PMC12927299; doi:10.1136/bmjopen-2025-103040)
Supplement: online supplemental file 1 [file bmjopen-16-2-s001.pdf]

Questionnaire

Development and Validation of a Knowledge, Attitude, and Practice (KAP) Questionnaire for Skin Cancer in the General Public: KAP-SC-Q

Social Demographics/Demografi Sosial/社会人口统计

Age:
Umur:
年龄：
(   ) years old/tahun/岁

Gender:
Jantina:
性别:
(   ) Male/Lelaki/男
(   ) Female/Perempuan/女

Ethnicity:
Bangsa:
种族:
(   ) Malay/Melayu/马来人
(   ) Chinese/Cina/华人
(   ) Indian/India/印度人
(   ) Others/Lain-lain/其他

When do you mainly stay outdoors?
Bilakah anda kebanyakannya berada di luar rumah?
您主要在什么时候呆在户外？
(   ) Early morning before 11am and/or late afternoon/evening (after 3pm)
Awal pagi sebelum 11 pagi dan/atau petang (selepas 3 petang)
早上11点前 和/或 下午3点过后
(   ) Midday (11am-3pm)
Tengah hari (11 pagi -3 petang)
中午（上午 11 点至下午 3 点）
(   ) All day (Sunrise to sunset)
Sepanjang hari (Matahari terbit hingga terbenam)
全天（日出到日落）
(   ) Not applicable
Tidak berkenaan
不适用

When going outdoors, how long are you exposed to the sun?
Apabila keluar rumah, berapa lama anda terdedah kepada matahari?

外出时，您会在阳光下暴晒多长时间？

( ) Less than one hour
Kurang dari 1 jam
不到一小时

( ) Between 1-3 hours per day
Antara 1-3 jam sehari
每天 1-3 小时

( ) Greater than 3 hours per day
Lebih dari 3 jam sehari
每天超过 3 小时

( ) Not applicable
Tidak berkenaan
不适用

Level of education:
Taraf Pendidikan Tertinggi:
最高学历:

( ) Primary education (UPSR)
Sekolah Rendah (UPSR)
小学 (UPSR)

( ) Secondary education (SPM, O Level)
Sekolah Menengah (SPM, O Level)
中学 (SPM, O Level)

( ) Tertiary education (Degree, Master, Doctorate)
Pengajian Tinggi (Ijazah Sarjana Muda, Ijazah Sarjana, Doktor Falsafah)
本科及以上 (学位、硕士、博士)

Living area:
Kawasan Tempat Tinggal:
生活区域:

( ) Urban/Bandar/城市
( ) Rural/Luar bandar/Kampung/乡村

Do you have a personal history of skin cancer?
Adakah anda mempunyai sejarah peribadi kanser kulit?
您有皮肤癌的个人病史吗？

( ) Yes/Ada/有
( ) No/Tiada/没有

Do you have a family history of skin cancer?
Adakah anda mempunyai sejarah keluarga yang menghidap kanser kulit?
您有皮肤癌家族史吗？

( ) Yes/Ada/有
( ) No/Tiada/没有

Do you have a diagnosis of skin disease? (e.g., eczema, acne, psoriasis, dermatitis)
Adakah anda mempunyai diagnosis penyakit kulit? (contoh: ekzema, jerawat, psoriasis, dermatitis)
你有皮肤病的诊断吗？（例如，湿疹、痤疮、牛皮癣、皮炎）

( ) Yes/Ada/有
( ) No/Tiada/没有

What is your skin tone?
Apakah ton kulit anda?
您的肤色是什么？

( ) Light/Cerah/苍白
( ) Fair/Putih/白皙
( ) Tan/Sawo matang/棕色
( ) Deep/Gelap/深色

What is your skin type?
Apakah jenis kulit anda?
您的皮肤类型是什么？

( ) **Type 1:** skin always burns, never tans, and is sensitive to UV exposure
Jenis 1: kulit sentiasa terbakar matahari, tidak pernah sawo matang, dan sensitif kepada pendedahan UV
类型 1: 皮肤经常灼伤，从不晒黑，并且对紫外线照射敏感

( ) **Type 2:** skin burns easily and tans minimally
Jenis 2: kulit mudah terbakar matahari dan sawo matang minima
类型 2: 皮肤容易灼伤，并且晒黑程度极低

( ) **Type 3:** skin burns moderately and tans gradually to light brown
Jenis 3: kulit terbakar matahari sederhana dan sawo matang secara beransur-ansur menjadi coklat muda
类型 3: 皮肤中度灼伤，并且逐渐晒成浅棕色

( ) **Type 4:** skin burns minimally and always tans well to moderately brown
Jenis 4: kulit terbakar matahari dengan minima dan sentiasa sawo matang hingga perang sederhana
类型 4: 皮肤轻微灼伤，并且总是晒黑至中度棕色

( ) **Type 5:** skin rarely burns and tans profusely to dark
Jenis 5: kulit jarang terbakar matahari dan sawo matang hingga gelap
类型 5: 皮肤很少灼伤，并且晒黑至深色

( ) **Type 6:** skin never burns, is deeply pigmented, and is least sensitive to UV exposure
Jenis 6: kulit tidak pernah terbakar matahari, berpigmen dalam, dan paling tidak sensitif terhadap pendedahan UV
类型 6: 皮肤从不灼伤，色素沉着，并且对紫外线照射最不敏感

How many moles do you have on the skin?
Berapa banyak tahi lalat yang anda ada pada kulit?
您全身大概有多少颗痣？

(   ) Low (0-50)
Kurang (0-50)
偏少 (0-50)
(   ) Moderate (51-100)
Sederhana (51-100)
中等 (51-100)
(   ) High (>100)
Banyak (>100)
偏多 (>100)

Do you have unusual moles (asymmetrical, irregular shape/border, uneven colour, diameter >6mm, growing moles)? ABCDE rule
Adakah anda mempunyai tahi lalat luar biasa (tak simetri, bentuk/sempadan tidak sekata, warna tidak sekata, diameter >6mm, tahi lalat tumbuh)? Peraturan ABCDE
您是否有不寻常的痣（不对称、形状/边界不规则、颜色不均匀、直径 >6 毫米、痣不断增大）？ ABCDE规则
(   ) Yes/Ada/有
(   ) No/Tiada/没有
(   ) Not applicable/Tidak berkenaan/不适用

Do you have any problem with hair loss or thinning?
Adakah anda mempunyai masalah rambut gugur atau menipis?
您有脱发或头发稀疏的问题吗？
(   ) Yes/Ada/有
(   ) No/Tiada/没有

Does your skin freckle?
Adakah kulit anda berjeragat?
您有雀斑吗？
(   ) Yes/Ada/有
(   ) No/Tiada/没有

Do you ever have a severe sunburn that blisters?
Adakah anda pernah mengalami selaran matahari yang teruk sehingga melepuh?
您是否曾被严重晒伤而起水泡？
(   ) Yes/Ada/有
(   ) No/Tiada/没有

Knowledge (Yes, No, Unsure)
Pengetahuan (Ya/Tidak/Tidak pasti)
知识 (是/否/不确定)

Statement/Kenyataan/问题	Yes/ Ya/ 是	No/Tidak /否	Unsure /Tidak pasti/ 不确定
**RISK FACTORS/FAKTOR RISIKO/风险因素**			
Ultraviolet (UV) radiation from the sun is the only cause of skin cancer.<br>Sinaran UV daripada matahari adalah salah satu faktor risiko kanser kulit.<br>来自太阳的紫外线辐射(UV)是导致皮肤癌的唯一原因。		X	
One of the risk factors for skin cancer includes those who are having lighter skin tone.<br>Salah satu faktor risiko kanser kulit termasuk orang yang mempunyai warna kulit yang lebih cerah.<br>肤色较浅是导致皮肤癌的其中一个危险因素。	X		
People with skin that burns or freckles easily are at a lower risk of getting skin cancer.<br>Orang yang mempunyai kulit yang mudah melecur atau berjeragat berisiko lebih rendah untuk kanser kulit.<br>皮肤容易灼伤或长雀斑的人患皮肤癌的风险较低。		X	
People who have a family and/or personal history of skin cancer are at increased risk of getting skin cancer.<br>Orang yang mempunyai keluarga dan/atau sejarah peribadi kanser kulit berisiko tinggi untuk mendapat kanser kulit.<br>有皮肤癌家族史和/或个人史的人患皮肤癌的风险增加。	X		
Sunburn accelerates skin ageing and is a leading cause in most cases of skin cancer.<br>Selaran matahari mempercepatkan penuaan kulit dan merupakan punca utama dalam kebanyakan kes kanser kulit.<br>晒伤会加速皮肤老化，并且是大多数皮肤癌病例的主要原因。	X		
Men are at greater risk to develop skin cancer.<br>Lelaki lebih berisiko untuk mendapat kanser kulit.<br>男性患皮肤癌的风险更大。	X		

Being bald or shaving your head raises the risk of getting skin cancer.<br>Menjadi botak atau mencukur kepala meningkatkan risiko mendapat kanser kulit.<br>秃顶或剃光头会增加患皮肤癌的风险。	X		
The risks of melanoma decrease with age.<br>Risiko melanoma berkurangan dengan usia.<br>黑色素瘤的风险会随着年龄的增长而降低。		X	
People with deeper skin tones are at lower risk of getting skin cancer.<br>Orang yang mempunyai warna kulit yang lebih gelap berisiko lebih rendah untuk mendapat kanser kulit.<br>肤色较深的人患皮肤癌的风险较低。		X	
**DIAGNOSIS/PENGESANAN/诊断**			
Skin cancer can be classified as melanoma and non-melanoma.<br>Kanser kulit boleh dikelaskan sebagai melanoma dan bukan melanoma.<br>皮肤癌可分为黑色素瘤和非黑色素瘤。	X		
Most skin cancers can be cured.<br>Kebanyakan kanser kulit boleh disembuhkan.<br>大多数皮肤癌都可以被治愈。	X		
Melanoma is the least common but is the most invasive skin cancer with the highest risk of death.<br>Melanoma adalah kanser kulit yang paling jarang berlaku tetapi merupakan kanser kulit yang paling invasif dengan risiko kematian tertinggi.<br>黑色素瘤最不常见，但却是侵袭性最强、死亡风险最高的一种皮肤癌。	X		
The incidence of melanoma is decreasing.<br>Insiden melanoma semakin berkurangan.<br>黑色素瘤的发病率正在下降。		X	
The first sign of melanoma is often a new mole or a change in the appearance of an existing mole.<br>Tanda pertama melanoma selalunya ialah tahi lalat baru atau perubahan pada rupa tahi lalat yang sedia ada.<br>黑色素瘤的第一个迹象通常是出现新的痣或现有痣的外观发生变化。	X		

Melanoma always develops in areas of your body that have exposure to the sun.<br>Melanoma sentiasa berkembang di kawasan badan yang terdedah di bawah matahari.<br>黑色素瘤总是出现在暴露在阳光下的身体部位。		**X**	
Early detection and treatment of melanoma have no effect on the curing and survival rate.<br>Pengesanan awal dan rawatan melanoma tidak mempunyai apa-apa kesan ke atas kadar penyembuhan dan kelangsungan hidup.<br>早期发现和治疗黑色素瘤对治愈率和存活率没有影响。		**X**	
**SCREENING/SARINGAN/筛查**			
A skin cancer screening is a visual exam of the skin that cannot be done by yourself and must be done by a health care provider.<br>Pemeriksaan kanser kulit ialah pemeriksaan visual kulit yang tidak boleh dilakukan sendiri dan mesti dilakukan oleh pakar penjagaan kesihatan.<br>皮肤癌筛查是一种皮肤视觉检查，不能由你自己完成，必须由医疗人员完成。		**X**	
A skin cancer screening checks the skin for moles, birthmarks, or other marks that are unusual in colour, size, shape, or texture.<br>Saringan kanser kulit memeriksa kulit untuk tahi lalat, tanda lahir atau tanda lain yang luar biasa dalam warna, saiz, bentuk atau tekstur.<br>皮肤癌筛查包含了检查皮肤是否有痣、胎记或其他颜色、大小、形状或质地异常的痕迹。	**X**		
Skin cancers can appear as a new, expanding, or changing growth, spot, or bump on the skin or a sore that bleeds and/or does not heal after several weeks.<br>Kanser kulit boleh muncul sebagai pertumbuhan, bintik atau benjolan baru, berkembang atau berubah pada kulit atau kudis yang berdarah dan/atau tidak sembuh selepas beberapa minggu.<br>皮肤癌可以显示为皮肤上新的、扩大的或变化的生长、斑点或肿块，或者几周后流血和/或不愈合的溃疡。	**X**		
A skin cancer screening rules out any moles that pose no danger at all or points out moles that could be dangerous or may already be cancerous.<br>Pemeriksaan kanser kulit mengecualikan mana-mana tahi lalat yang tidak membawa bahaya dan juga menunjukkan tahi lalat yang boleh membawa bahaya atau sudah menjadi kanser.	**X**		

皮肤癌筛查会排除任何根本不会造成危险的痣，或指出可能有危险或可能已经癌变的痣。			
A visual check of your skin can confirm the diagnosis of skin cancer.<br>Pemeriksaan visual kulit boleh mengesahkan diagnosis kanser kulit.<br>通过目视检查你自己的皮肤可以直接诊断出皮肤癌。		X	
A skin cancer bump or lesion usually heals on its own without any intervention.<br>Benjolan atau lesi kanser kulit biasanya sembuh dengan sendirinya tanpa sebarang rawatan.<br>皮肤癌肿块或病变通常会自行愈合，无需任何干预。		X	
If the doctor thinks that a suspicious area might be skin cancer, he may start you on treatment right away to prevent cancerous cells from spreading to other parts of the body.<br>Jika doktor berpendapat bahawa kawasan yang mencurigakan mungkin kanser kulit, dia akan memulakan rawatan dengan segera untuk mengelakkan sel-sel kanser daripada merebak ke bahagian badan yang lain.<br>如果医生认为可疑部位可能是皮肤癌，他会立即开始治疗，以防止癌细胞扩散到身体的其他部位。		X	
A skin biopsy can help diagnose skin cancer.<br>Biopsi kulit boleh membantu mendiagnosis kanser kulit.<br>皮肤活检可以帮助诊断皮肤癌。	X		
**PREVENTION/PENCEGAHAN/预防**			
Wearing long sleeve clothes and using sunblock with SPF15 can reduce the risk of getting skin cancer.<br>Memakai pakaian lengan panjang dan menggunakan pelindung matahari dengan SPF15 boleh mengurangkan risiko mendapat kanser kulit.<br>穿长袖衣服和使用 SPF15的防晒霜可以降低患皮肤癌的风险。		X	
Using an umbrella has no effect in preventing skin cancer.<br>Menggunakan payung tidak mempunyai kesan untuk mencegah kanser kulit.<br>使用雨伞对预防皮肤癌没有作用。		X	
You only need to wear sunscreen on sunny days.<br>Anda hanya perlu memakai pelindung matahari pada hari yang cerah		X	

你只需要在晴天涂防晒霜。			
Sunscreen with SPF30 means that you can stay in the sun for 30 minutes without burning.<br>Pelindung matahari dengan SPF30 bermakna anda boleh berada di bawah sinar matahari selama 30 minit tanpa selaran matahari<br>SPF30的防晒霜意味着你可以在阳光下停留30分钟而不会被晒伤。		X	
Seeking shade between 10 a.m. and 4 p.m. when the sun's Ultraviolet rays are strongest can help to prevent skin cancer.<br>Mencari tempat teduh antara 10 pagi dan 4 petang apabila sinaran UV matahari paling kuat boleh membantu mencegah kanser kulit.<br>上午 10 点到下午 4 点之间是太阳的紫外线最强的时候，这时候寻找阴凉处可以帮助预防皮肤癌。	X		
Supplements that contain antioxidants such as vitamin A, C, and E might be a protective strategy to reduce the risk of getting skin cancer.<br>Suplemen yang mengandungi antioksidan seperti vitamin A, C dan E merupakan salah satu strategi yang boleh mengurangkan risiko untuk mendapat kanser kulit.<br>含有抗氧化剂（如维生素 A、C 和 E）的营养品可以降低患皮肤癌的风险。	X		
**Yes/Ya/是= 16**<br>**No/Tidak/否= 15**<br>**Total/Tidak pasti/总共= 31**			

Attitude (Strongly disagree, Disagree, Uncertain, Agree, Strongly agree)

Sikap (Sangat tidak setuju, Tidak setuju, Tidak pasti, Setuju, Sangat setuju)

态度 （非常不同意，不同意，不确定，同意，非常同意）

Please read the following statements carefully and indicate how much you agree or disagree based on your own situation.
Sila baca soalan berikut dengan teliti dan nyatakan sejauh mana anda bersetuju atau tidak bersetuju berdasarkan situasi anda sendiri.

请认真阅读以下问题，根据自己的实际情况表明您同意或不同意的程度。

**Statement**<br>**Kenyataan**<br>问题	**Strongly disagree**<br>**Sangat tidak setuju**非常不同意	**Disagree**<br>**Tidak setuju**<br>不同意	**Uncertain**<br>**Tidak pasti**<br>不确定	**Agree**<br>**Setuju**<br>同意	**Strongly agree**<br>**Sangat setuju**<br>非常同意
**RISK FACTORS**<br>**Risiko**<br>风险因素					
Skin cancer is a skin problem that can cause death.<br>Kanser kulit adalah masalah kulit yang boleh menyebabkan kematian.<br>皮肤癌是一种可导致死亡的皮肤问题。				**X**	
Skin cancer is totally incurable.<br>Kanser kulit tidak boleh diubati.<br>皮肤癌是完全无法治愈的。		**X**			
Skin cancer is the mutation of the cells in the skin that cause abnormal skin texture.<br>Kanser kulit ialah mutasi sel dalam kulit yang menyebabkan tekstur kulit yang tidak normal.<br>皮肤癌是导致皮肤纹理异常的皮肤细胞突变。				**X**	
Skin cancer is highly preventable.<br>Kanser kulit boleh dicegah.<br>皮肤癌是高度可预防的。				**X**	
The number of moles will not affect the risk of getting skin cancer.<br>Jumlah tahi lalat tidak akan menjejaskan risiko mendapat kanser kulit.<br>痣的数量不会影响患皮肤癌的风险。		**X**			

Melanoma skin cancer risk is around doubled in people with freckles.<br>Risiko kanser kulit melanoma adalah dua kali ganda bagi orang yang mempunyai bintik-bintik.<br>有雀斑的人患黑色素瘤皮肤癌的风险大约增加一倍。				**X**	
Melanoma skin cancer risk is up to three times as high in people with dark-haired people, compared with red/red-blonde hair.<br>Risiko kanser kulit melanoma adalah sehingga tiga kali lebih tinggi pada orang yang berambut gelap, berbanding dengan rambut merah/merah perang.<br>与红色/红色金发的人相比，黑头发的人患黑色素瘤皮肤癌的风险高达三倍。		**X**			
Melanoma skin cancer risk is increased regardless of whether sunburn occurred in childhood or adulthood.<br>Risiko kanser kulit melanoma meningkat tanpa mengira sama ada selaran matahari berlaku pada zaman kanak-kanak atau dewasa.<br>无论晒伤发生在童年还是成年，黑色素瘤皮肤癌的风险都会增加。				**X**	
The causes of skin cancer are too much exposure to sunlight and UV and using too many cosmetic products for whitening.<br>Punca-punca kanser kulit adalah terlalu banyak terdedah kepada cahaya matahari dan UV serta menggunakan terlalu banyak produk kosmetik untuk pemutihan.<br>患皮肤癌的原因是过多地暴露在阳光和紫外线下，以及使用过多的美白化妆品。				**X**	

Skin whitening cosmetic products are not one of the main causes of skin cancer.<br>Produk kosmetik pemutih kulit bukanlah salah satu punca utama kanser kulit.<br>皮肤美白化妆品不是导致皮肤癌的主要原因之一。		**X**			
Taking medications that suppress or weaken your immune system will not increase your risk of skin cancer.<br>Pengambilan ubat-ubatan yang menekan atau melemahkan sistem imun anda tidak akan meningkatkan risiko mendapat kanser kulit.<br>服用抑制或削弱免疫系统的药物不会增加患皮肤癌的风险。		**X**			
People who have many moles or abnormal moles are at an increased risk of skin cancer.<br>Orang yang mempunyai banyak tahi lalat atau tahi lalat yang tidak normal berisiko tinggi mendapat kanser kulit.<br>有很多痣或痣异常的人会增加患皮肤癌的风险。		**X**			
A suntan is a sign of being healthy.<br>Berjemur adalah tanda sihat.<br>晒黑是健康的标志。		**X**			
Geographic location is not related to the increased risk of skin cancer.<br>Lokasi geografi tidak berkaitan dengan peningkatan risiko kanser kulit.<br>地理位置与皮肤癌风险增加无关。		**X**			
If one of your parents or a sibling has had skin cancer, you may have an increased risk of the disease.				**X**	

Jika salah seorang ibu bapa atau adik-beradik anda menghidap kanser kulit, anda mungkin mempunyai peningkatan risiko penyakit itu.<br>如果您的父母或兄弟姐妹之一患有皮肤癌，您患该病的风险可能会增加。					
**DIAGNOSIS/DETECTION**<br>**DIAGNOSIS / PENGESANAN**<br>**诊断/检测**					
When I see any new mole or any change in the mole, I think there is no need to consult with the doctor.Apabila saya melihat apa-apa tahi lalat baru atau apa-apa perubahan pada tahi lalat, saya rasa tidak perlu berunding dengan doktor.<br>当我看到任何新的痣或痣有任何变化时，我认为没有必要咨询医生。		**X**			
I have regular skin examinations with a dermatologist to screen for skin cancer.<br>Saya kerap menjalani pemeriksaan kulit dengan pakar dermatologi untuk menyaring kanser kulit.<br>我与皮肤科医生定期进行皮肤检查以筛查皮肤癌。				**X**	
I do not examine my skin often for new skin growths or changes in existing moles, freckles, bumps and birthmarks.<br>Saya tidak kerap memeriksa kulit saya untuk ketumbuhan kulit baru atau perubahan pada tahi lalat, jeragat, benjolan dan tanda lahir yang sedia ada.<br>我不会经常检查我的皮肤是否有新的皮肤生长或现有痣、雀斑、肿块和胎记的变化。		**X**			

Individuals with dark skin are not at risk of incidence of skin cancer.<br>Individu yang berkulit gelap tidak berisiko mendapat kanser kulit.<br>皮肤黝黑的人没有患皮肤癌的风险。		**X**			
Individuals with dark skin require the same protection and regular examination needed for those with lighter skin types.<br>Individu yang berkulit gelap memerlukan perlindungan yang sama dan pemeriksaan tetap yang diperlukan bagi mereka yang mempunyai jenis kulit yang lebih cerah.<br>深色皮肤的人同样需要与浅色皮肤类型的人有相同的保护和定期检查。				**X**	
**SCREENING TOOL**<br>**SARINGAN**<br>**检查**					
Observing any abnormalities, rashes, or inflammation on the skin are the screening method for skin cancer.<br>Memerhati sebarang keabnormalan, ruam, atau keradangan pada kulit adalah kaedah saringan untuk kanser kulit.<br>观察皮肤有无异常、皮疹或炎症，是皮肤癌的筛查方法。				**X**	
Skin cancer screenings may be done by yourself, your primary care provider, or a dermatologist.<br>Pemeriksaan kanser kulit boleh dilakukan oleh anda sendiri, pembekal penjagaan utama anda atau pakar dermatologi.<br>皮肤癌筛查可由您自己、您的初级保健提供者或皮肤科医生进行。				**X**	

Screening for skin cancer can only be done by the health care provider.<br>Saringan untuk kanser kulit hanya boleh dilakukan oleh penyedia penjagaan kesihatan.<br>皮肤癌筛查只能由医疗保健提供者进行。		**X**			
**PREVENTION**<br>**PENCEGAHAN**<br>**预防**					
Sun protection is important to reduce the risk of skin cancer.<br>Perlindungan matahari adalah penting untuk mengurangkan risiko kanser kulit.<br>防晒对于降低患皮肤癌的风险很重要。				**X**	
Sunscreen is only necessary on sunny days.<br>Pelindung matahari hanya diperlukan pada hari yang cerah.<br>只有在晴天才需要防晒霜。		**X**			
Sun protection is a complicated and expensive process to conduct.<br>Perlindungan matahari adalah proses yang rumit dan mahal untuk dijalankan.<br>防晒是一个复杂而昂贵的过程。		**X**			
Sun protection is important while driving in the daytime.<br>Perlindungan matahari adalah penting semasa memandu pada waktu siang.<br>白天开车防晒很重要。				**X**	
Sunscreen is required on a cloudy or rainy day.<br>Pelindung matahari diperlukan pada hari mendung atau hujan.<br>阴天或雨天都需要涂防晒霜。				**X**	

Sunscreen should not be applied when going for outdoor activities on a cloudy day.<br>Pelindung matahari tidak boleh digunakan semasa melakukan aktiviti luar pada hari yang mendung.<br>阴天外出活动时不宜涂抹防晒霜。		X			
Sunscreen should be applied when going for outdoor activities on a sunny day.<br>Pelindung matahari harus digunakan apabila pergi untuk aktiviti luar pada hari yang cerah.<br>晴天外出活动时应涂抹防晒霜。				X	
Sunscreen should be applied when going swimming at the pool, beach, or waterfall.<br>Pelindung matahari hendaklah digunakan semasa berenang di kolam renang, pantai atau air terjun.<br>在游泳池、海滩或瀑布游泳时应涂抹防晒霜。				X	
Sunscreen should be applied when attending any occasion at night.<br>Pelindung matahari hendaklah digunakan apabila menghadiri sebarang majlis pada waktu malam.<br>晚上出席任何场合都要涂抹防晒霜。		X			
Avoiding excessive exposure to sunlight, wearing sunscreen when going out, and covering most body parts are the best preventive measures.<br>Elakkan pendedahan yang berlebihan kepada cahaya matahari, memakai pelindung matahari semasa keluar rumah, dan menutup kebanyakan bahagian				X	

badan adalah langkah pencegahan terbaik.<br>避免过度暴露在阳光下，外出时涂抹防晒霜,并且遮盖大部分身体部位是最好的预防措施。					
Sunbathing is good for my skin.<br>Berjemur adalah baik untuk kulit saya.<br>日光浴对我的皮肤有好处。		X			
I take antioxidant-rich foods such as carrots and tomatoes to prevent skin cancer.<br>Saya makan makanan yang kaya dengan antioksidan seperti lobak merah dan tomato untuk mengelakkan kanser kulit.<br>我吃富含抗氧化剂的食物，比如胡萝卜和西红柿，以预防皮肤癌。				X	
**TOTAL 35**<br>**AGREE = 18, DISAGREE = 17**					

Practice (Never, Rarely, Sometimes, Often, Always)

Amalan (Tidak Pernah, Jarang, Kadang-Kadang,Selalu,Sentiasa)

行为 (没有，不常，有时，经常，时刻)

Statement<br>Kenyataan<br>问题	Never<br>Tidak Pernah<br>没有	Rarely<br>Jarang<br>不常	Sometimes<br>Kadang-Kadang<br>有时	Often<br>Selalu<br>经常	Always<br>Sentiasa<br>时刻
**DIAGNOSIS/ DETECTION**<br>**DIAGNOSIS/ PENGESANAN**<br>**诊断/检测**					
I examine my skin (head to toe) thoroughly annually.				X	

Saya memeriksa kulit saya (kepala hingga kaki) dengan teliti setiap tahun.<br><br>我每年都会彻底检查我的皮肤（从头到脚）。					
I see a skin specialist at least once a year for a professional skin exam.<br><br>Saya berjumpa pakar kulit sekurang-kurangnya sekali setahun untuk peperiksaan kulit profesional.<br><br>我每年至少看一次皮肤专家进行专业皮肤检查。					**X**
**SCREENING**<br>**SARINGAN KESIHATAN**<br>**检查**					
I do the skin biopsy test every 2 years.<br><br>Saya melakukan ujian biopsi kulit setiap 2 tahun.<br><br>我每 2 年进行一次皮肤活检。	**X**				
I do the skin exam with health care professionals to see whether there are abnormalities in my skin (colour, shape, texture).<br><br>Saya melakukan pemeriksaan kulit dengan profesional penjagaan kesihatan untuk melihat ada keabnormalan pada kulit saya (warna, bentuk, tekstur) atau tidak.				**X**	

我去看医疗保健专业人员进行检查，看看我的皮肤是否有异常（颜色、形状、质地）。					
**SELF DIAGNOSIS**<br>**DIAGNOSIS SENDIRI**<br>**自己的诊断**					
I ignore the worrisome change.<br><br>Saya mengabaikan perubahan yang membimbangkan.<br><br>我忽略了令人担忧的皮肤变化。	**X**				
I will self-examine the skin and check for abnormal signs.<br><br>Saya akan memeriksa kulit sendiri dan menyemak ada tanda-tanda yang abnormal atau tidak.<br><br>我会自行检查皮肤并且检查是否有异常迹象。					**X**
**HAT / UMBRELLA**<br>**TUDUNG / PAYUNG**<br>**帽子或者雨伞**					
I wear the hat with a wide brim (all around) to shade my face, head, ears, and neck when I go outside.<br><br>Saya memakai topi dengan tepi lebar (di sekeliling) untuk memayungi muka, kepala, telinga dan leher saya apabila saya keluar.					**X**

当我出门时，我戴着宽边（四周）的帽子来遮挡我的脸、头、耳朵和脖子。					
I stay under a patio umbrella which is made up of sunbrella when having tea with friends outside the cafe.<br><br>Saya duduk di bawah payung patio yang terdiri daripada sunbrella apabila menjamu selera bersama rakan-rakan di luar kafe.<br><br>在咖啡馆外和朋友喝茶时，我会呆在遮阳伞下。				X	
**SUNSCREEN**<br>**PELINDUNG MATAHARI**<br>**防晒霜**					
I apply sunscreen 30 minutes before I plan to go out and when outdoors, reapply sunscreen every 2 hours.<br><br>Saya menggunakan pelindung matahari 30 minit sebelum saya merancang untuk keluar. Apabila berada di luar, Saya menggunakan semula pelindung matahari setiap 2 jam.<br><br>在外出的30 分钟前，我会涂抹防晒霜，然后在户外时，每 2 小时重新涂抹一次防晒霜。					X
I do not reapply sunscreen after excessive swimming or excessive sweating.	X				

Saya tidak menggunakan semula pelindung matahari selepas berenang atau berpeluh berlebihan.<br><br>过度游泳或过度出汗后，我不会重新涂抹防晒霜。					
I use a stronger (UVA/UVB) sunscreen with a sun protection factor (SPF 50) or higher.<br><br>Saya menggunakan pelindung matahari yang lebih kuat dengan faktor perlindungan matahari (SPF 50) atau lebih tinggi.<br><br>我使用防晒系数 (SPF 50) 或更高和更强 的防晒霜。				**X**	
I just apply sunscreen without checking its expiry date.<br>Saya menggunakan pelindung matahari tanpa menyemak tarikh luputnya.<br><br>我只是涂防晒霜而且重来不检查其有效期。	**X**				
I avoid sunburn by applying sunscreen to reduce the risk of skin cancer.<br><br>Saya mengelakkan selaran matahari dengan menggunakan pelindung matahari untuk mengurangkan risiko kanser kulit.					**X**

我通过涂抹防晒霜来避免晒伤，以及降低得到皮肤癌症的风险。					
I use sunscreen in the form of lotion instead of spray.<br><br>Saya menggunakan pelindung matahari dalam bentuk losyen bukannya semburan.<br><br>我使用乳液式的防晒霜而不是喷雾式的。	**X**				
I apply a two finger rule which is by squeezing a small amount to the palm and spread to the tip of the index and middle fingers when applying sunscreen for the full body.<br><br>Saya menggunakan peraturan dua jari iaitu picit sedikit ke tapak tangan dan sebarkan ke hujung telunjuk apabila menggunakan pelindung matahari kepada seluruh badan.<br><br>涂抹全身防晒霜时，我采用两指规则，即是在手掌上挤少量，然后涂抹到食指和中指的尖端。				**X**	
**CLOTHING**<br>**PAKAIAN**<br>**服装**					
I wear singlets when going outside.	**X**				

Saya memakai singlet apabila keluar.<br><br>我穿背心出门。					
I wear UV-protective clothing when I go outside.<br><br>Saya memakai pakaian pelindung UV apabila keluar.<br><br>外出时，我穿防紫外线的衣服。				**X**	
I wear clothing made up of fabric (polyester, nylon, wool) to prevent UV rays.<br><br>Saya memakai pakaian yang diperbuat daripada fabrik (poliester, nilon) untuk mengelakkan sinaran UV.<br><br>我穿由(尼龙、羊毛）制成的衣服来防止紫外线。					**X**
I wear more layers of clothing to prevent UV rays when I go hiking and jogging at parks and beaches.<br><br>Saya memakai lebih banyak lapisan pakaian untuk mengelakkan sinaran UV apabila saya pergi berjoging di taman dan pantai.				**X**	

当我去公园和海滩远足和慢跑时，我会穿更多的衣服来防止紫外线。					
I wear bright-coloured clothing when I go outside.<br><br>Saya memakai pakaian yang berwarna terang apabila keluar.<br><br>外出时,我穿颜色鲜艳的衣服。		X			
**SWIMMING (SEA/POOL)<br>BERENANG (LAUT/KOLAM RENANG)<br>游泳 （海边或游泳池）**					
I like to swim during the noon time.<br><br>Saya suka berenang pada waktu tengah hari.<br><br>我喜欢在中午时间游泳。	X				
I reapply sunscreen every 40-80 minutes when swimming.<br><br>Saya menggunakan semula pelindung matahari setiap 40-80 minit apabila berenang.<br><br>当我游泳时，我每 40-80 分钟会重新涂抹一次防晒霜。				X	
I do not reapply sunscreen after towelling.<br><br>Saya tidak menggunakan semula pelindung matahari	X				

selepas memakai tuala untuk mengelap badan.<br><br>当我用毛巾擦完身体后，我不会重新涂抹防晒霜。					
I wear a long swimming suit when swimming.<br><br>Saya memakai baju renang yang panjang ketika berenang.<br><br>我穿长泳衣去游泳。					X
For water-resistant activity (swimming), I apply (UVA/UVB) sunscreen with an SPF of 50 or higher.<br><br>Untuk aktiviti kalis air (berenang), saya menggunakan pelindung matahari SPF 50 atau lebih tinggi.<br><br>对于防水活动（游泳），我使用 SPF 为 50 或更高的 防晒霜。					X
**WORKING HABITS**<br>**TABIAT PERKERJAAN**<br>**工作或日常习惯**					
I spend most of my time playing with kids outside like in the garden or playground for an hour or two.<br><br>Saya menghabiskan sebahagian masa bermain dengan kanak-kanak di luar seperti di taman permainan selama satu atau dua jam.	X				

大部分时间, 我都在外面（比如花园或操场）和孩子们一起玩耍至少 1个小时以上。					
I do not choose my work area next to the window in the office.<br><br>Saya tidak memilih kawasan kerja saya di sebelah tingkap di pejabat.<br><br>在办公室里，我不会选择窗户旁边的工作区域。					X
I help remove any debris at the construction site for 1-2 hours without using any sun protection things.<br><br>Saya membuang serpihan di tapak pembinaan selama 1-2 jam tanpa menggunakan pelindung matahari.<br><br>当我帮助清除建筑工地的任何杂物 1-2 小时，我不使用任何防晒用品。	X				
I apply sunscreen before planting and harvesting the crops for 1-2 hours every day.<br><br>Saya menggunakan pelindung matahari sebelum menanam dan menuai tanaman selama 1-2 jam setiap hari.<br><br>我每天在播种和收割农作物前， 我都会涂抹防晒霜 。					X

Correct - 18
Incorrect - 11
Total - 29
