# Supplementary material for: Knowledge, attitudes and practices toward skin cancer prevention among Malaysian adults: a cross-sectional online survey
Source: BMJ Open. 2026 Feb 22;16(2):e103040. doi: 10.1136/bmjopen-2025-103040 (PMC12927299; doi:10.1136/bmjopen-2025-103040)
Supplement: online supplemental file 2 [file bmjopen-16-2-s002.docx]

Appendix

**Table Summary of Psychometric Properties of the Skin Cancer KAP Questionnaire**

| **Construct** | **No. of Items** | **Measurement Focus** | **Example of Item Type** | **Internal Consistency (Cronbach’s α)** |
| --- | --- | --- | --- | --- |
| Knowledge | 30 | Risk factors, symptoms, UV exposure, preventive strategies | “Sunburn increases the risk of skin cancer.” (True/False) | 0.81 |
| Attitudes | 32 | Perceptions and beliefs regarding screening, prevention, UV protection | “Using sunscreen is essential to prevent skin cancer.” (Likert scale) | 0.86 |
| Practices | 29 | Protective behaviours (sun avoidance, sunscreen use, skin examination) | “How often do you apply sunscreen when outdoors?” | 0.84 |
