# Supplementary material for: Knowledge, attitudes and practices toward skin cancer prevention among Malaysian adults: a cross-sectional online survey
Source: BMJ Open. 2026 Feb 22;16(2):e103040. doi: 10.1136/bmjopen-2025-103040 (PMC12927299; doi:10.1136/bmjopen-2025-103040)
Supplement: online supplemental file 3 [file bmjopen-16-2-s003.docx]

**Appendices**

**Table S1 Knowledge of participants towards skin cancer**

| **Statement** | **n (%)** |
| --- | --- |
| Ultraviolet (UV) radiation from the sun is the only cause of skin cancer. | |
| Yes | 160 (41.45) |
| No | 154 (39.90) |
| Unsure | 72 (18.65) |
| One of the risk factors for skin cancer includes those who are having lighter skin tone. | |
| Yes | 155 (40.16) |
| No | 105 (27.20) |
| Unsure | 126 (32.64) |
| People with skin that burns or freckles easily are at a lower risk of getting skin cancer. | |
| Yes | 72 (18.65) |
| No | 151 (39.12) |
| Unsure | 163 (42.23) |
| People who have a family and/or personal history of skin cancer are at increased risk of getting skin cancer. | |
| Yes | 308 (79.79) |
| No | 35 (9.07) |
| Unsure | 43 (11.14) |
| Sunburn accelerates skin ageing and is a leading cause in most cases of skin cancer. | |
| Yes | 268 (69.43) |
| No | 41 (10.62) |
| Unsure | 77 (19.95) |
| Men are at greater risk to develop skin cancer. | |
| Yes | 81 (20.98) |
| No | 109 (28.24) |
| Unsure | 196 (50.78) |
| Being bald or shaving your head raises the risk of getting skin cancer. | |
| Yes | 125 (32.38) |
| No | 124 (32.12) |
| Unsure | 137 (35.49) |
| The risks of melanoma decrease with age. | |
| Yes | 76 (19.69) |
| No | 153 (39.64) |
| Unsure | 157 (40.67) |
| People with deeper skin tones are at lower risk of getting skin cancer. | |
| Yes | 128 (33.16) |
| No | 122 (31.61) |
| Unsure | 136 (35.23) |
| Skin cancer can be classified as melanoma and non-melanoma. | |
| Yes | 198 (51.30) |
| No | 22 (5.70) |
| Unsure | 166 (43.01) |
| Most skin cancers can be cured. | |
| Yes | 179 (46.37) |
| No | 53 (13.73) |
| Unsure | 154 (39.90) |
| Melanoma is the least common but is the most invasive skin cancer with the highest risk of death. | |
| Yes | 142 (36.79) |
| No | 38 (9.84) |
| Unsure | 206 (53.37) |
| The incidence of melanoma is decreasing. | |
| Yes | 75 (19.43) |
| No | 88 (22.80) |
| Unsure | 223 (57.77) |
| The first sign of melanoma is often a new mole or a change in the appearance of an existing mole. | |
| Yes | 188 (48.70) |
| No | 35 (9.07) |
| Unsure | 163 (42.23) |
| Melanoma always develops in areas of your body that have exposure to the sun. | |
| Yes | 172 (44.56) |
| No | 52 (13.47) |
| Unsure | 162 (41.97) |
| Early detection and treatment of melanoma have no effect on the curing and survival rate. | |
| Yes | 85 (22.02) |
| No | 185 (47.93) |
| Unsure | 116 (30.05) |
| A skin cancer screening is a visual exam of the skin that cannot be done by yourself and must be done by a health care provider. | |
| Yes | 240 (62.18) |
| No | 66 (17.10) |
| Unsure | 80 (20.73) |
| A skin cancer screening checks the skin for moles, birthmarks, or other marks that are unusual in colour, size, shape, or texture. | |
| Yes | 273 (70.73) |
| No | 21 (5.44) |
| Unsure | 92 (23.83) |
| Skin cancers can appear as a new, expanding, or changing growth, spot, or bump on the skin or a sore that bleeds and/or does not heal after several weeks. | |
| Yes | 244 (63.21) |
| No | 23 (5.96) |
| Unsure | 119 (30.83) |
| A skin cancer screening rules out any moles that pose no danger at all or points out moles that could be dangerous or may already be cancerous. | |
| Yes | 221 (57.25) |
| No | 50 (12.95) |
| Unsure | 115 (29.79) |
| A visual check of your skin can confirm the diagnosis of skin cancer. | |
| Yes | 85 (22.02) |
| No | 191 (49.48) |
| Unsure | 110 (28.50) |
| A skin cancer bump or lesion usually heals on its own without any intervention. | |
| Yes | 50 (12.95) |
| No | 210 (54.40) |
| Unsure | 126 (32.64) |
| If the doctor thinks that a suspicious area might be skin cancer, he may start you on treatment right away to prevent cancerous cells from spreading to other parts of the body. | |
| Yes | 218 (56.48) |
| No | 66 (17.10) |
| Unsure | 102 (26.42) |
| A skin biopsy can help diagnose skin cancer. | |
| Yes | 275 (71.24) |
| No | 20 (5.18) |
| Unsure | 91 (23.58) |
| Wearing long sleeve clothes and using sunblock with SPF15 can reduce the risk of getting skin cancer. | |
| Yes | 284 (73.58) |
| No | 49 (12.69) |
| Unsure | 53 (13.73) |
| Using an umbrella has no effect in preventing skin cancer. | |
| Yes | 79 (20.47) |
| No | 232 (60.10) |
| Unsure | 75 (19.43) |
| You only need to wear sunscreen on sunny days. | |
| Yes | 75 (19.43) |
| No | 261 (67.62) |
| Unsure | 50 (12.95) |
| Sunscreen with SPF30 means that you can stay in the sun for 30 minutes without burning. | |
| Yes | 103 (26.68) |
| No | 197 (51.04) |
| Unsure | 86 (22.28) |
| Seeking shade between 10 a.m. and 4 p.m. when the sun’s ultraviolet rays are strongest can help to prevent skin cancer. | |
| Yes | 276 (71.50) |
| No | 63 (16.32) |
| Unsure | 47 (12.18) |
| Supplements that contain antioxidants such as vitamin A, C, and E might be a protective strategy to reduce the risk of getting skin cancer. | |
| Yes | 264 (68.39) |
| No | 44 (11.40) |
| Unsure | 78 (20.21) |

**Table S2 Attitudes of participants towards skin cancer**

| **No.** | **Statement** | **Strongly disagree**  **n (%)** | **Disagree**  **n (%)** | **Unsure**  **n (%)** | | **Agree**  **n (%)** | **Strongly agree**  **n (%)** |
| --- | --- | --- | --- | --- | --- | --- | --- |
| 1 | Skin cancer is a skin problem that can cause death. | 14 (3.63) | 14 (3.63) | 67 (17.36) | 234 (60.62) | | 57 (14.77) |
| 2 | Skin cancer is totally incurable. | 30 (7.77) | 176 (45.60) | 106 (27.46) | 65 (16.84) | | 9 (2.33) |
| 3 | Skin cancer is the mutation of the cells in the skin that cause abnormal skin texture. | 5 (1.30) | 12 (3.11) | 101 (26.17) | 210 (54.40) | | 58 (15.03) |
| 4 | Skin cancer is highly preventable. | 6 (1.55) | 22 (5.70) | 71 (18.39) | 223 (57.77) | | 64 (16.58) |
| 5 | The number of moles will not affect the risk of getting skin cancer. | 12 (3.11) | 83 (21.50) | 170 (44.04) | 106 (27.46) | | 15 (3.89) |
| 6 | Melanoma skin cancer risk is around doubled in people with freckles. | 12 (3.11) | 51 (13.21) | 188 (48.70) | 130 (33.68) | | 5 (1.30) |
| 7 | Melanoma skin cancer risk is up to three times as high in people with dark-haired people, compared with red/red-blonde hair. | 22 (5.70) | 85 (22.02) | 191 (49.48) | 82 (21.24) | | 6 (1.55) |
| 8 | Melanoma skin cancer risk is increased regardless of whether sunburn occurred in childhood or adulthood. | 8 (2.07) | 31 (8.03) | 139 (36.01) | 188 (48.70) | | 20 (5.18) |
| 9 | The causes of skin cancer are too much exposure to sunlight and UV and using too many cosmetic products for whitening. | 8 (2.07) | 37 (9.59) | 119 (30.83) | 185 (47.93) | | 37 (9.59) |
| 10 | Skin whitening cosmetic products are not one of the main causes of skin cancer. | 17 (4.40) | 92 (23.83) | 138 (35.75) | 125 (32.38) | | 14 (3.63) |
| 11 | Taking medications that suppress or weaken your immune system will not increase your risk of skin cancer. | 29 (7.51) | 124 (32.12) | 128 (33.16) | 90 (23.32) | | 15 (3.89) |
| 12 | People who have many moles or abnormal moles are at an increased risk of skin cancer. | 9 (2.33) | 33 (8.55) | 140 (36.27) | 174 (45.08) | | 30 (7.77) |
| 13 | A suntan is a sign of being healthy. | 37 (9.59) | 151 (39.12) | 108 (27.98) | 80 (20.73) | | 10 (2.59) |
| 14 | Geographic location is not related to the increased risk of skin cancer. | 51 (13.21) | 138 (35.75) | 101 (26.17) | 84 (21.76) | | 12 (3.11) |
| 15 | If one of your parents or a sibling has had skin cancer, you may have an increased risk of the disease. | 9 (2.33) | 30 (7.77) | 68 (17.62) | 228 (59.07) | | 51 (13.21) |
| 16 | When I see any new mole or any change in the mole, I think there is no need to consult with the doctor. | 56 (14.51) | 144 (37.31) | 85 (22.02) | 91 (23.58) | | 10 (2.59) |
| 17 | I have regular skin examinations with a dermatologist to screen for skin cancer. | 45 (11.66) | 74 (19.17) | 80 (20.73) | 156 (40.41) | | 31 (8.03) |
| 18 | I do not examine my skin often for new skin growths or changes in existing moles, freckles, bumps and birthmarks. | 24 (6.22) | 100 (25.91) | 71 (18.39) | 154 (39.90) | | 37 (9.59) |
| 19 | Individuals with dark skin are not at risk of incidence of skin cancer. | 62 (16.06) | 178 (46.11) | 96 (24.87) | 46 (11.92) | | 4 (1.04) |
| 20 | Individuals with dark skin require the same protection and regular examination needed for those with lighter skin types. | 7 (1.81) | 19 (4.92) | 75 (19.43) | 222 (57.51) | | 63 (16.32) |
| 21 | Observing any abnormalities, rashes, or inflammation on the skin are the screening method for skin cancer. | 5 (1.30) | 20 (5.18) | 100 (25.91) | 224 (58.03) | | 37 (9.59) |
| 22 | Skin cancer screenings may be done by yourself, your primary care provider, or a dermatologist. | 7 (1.81) | 41 (10.62) | 98 (25.39) | 204 (52.85) | | 36 (9.33) |
| 23 | Screening for skin cancer can only be done by the health care provider. | 3 (0.78) | 87 (22.54) | 101 (26.17) | 162 (41.97) | | 33 (8.55) |
| 24 | Sun protection is important to reduce the risk of skin cancer. | 5 (1.30) | 10 (2.59) | 78 (20.21) | 204 (52.85) | | 89 (23.06) |
| 25 | Sunscreen is only necessary on sunny days. | 82 (21.24) | 165 (42.75) | 59 (15.28) | 73 (18.91) | | 7 (1.81) |
| 26 | Sun protection is a complicated and expensive process to conduct. | 37 (9.59) | 158 (40.93) | 66 (17.10) | 115 (29.75) | | 10 (2.59) |
| 27 | Sun protection is important while driving in the daytime. | 6 (1.55) | 25 (6.48) | 55 (14.25) | 204 (52.85) | | 96 (24.87) |
| 28 | Sunscreen is required on a cloudy or rainy day. | 7 (1.81) | 64 (16.58) | 92 (23.83) | 158 (40.93) | | 65 (16.84) |
| 29 | Sunscreen should not be applied when going for outdoor activities on a cloudy day. | 63 (16.32) | 156 (40.41) | 83 (21.50) | 79 (20.47) | | 5 (1.30) |
| 30 | Sunscreen should be applied when going for outdoor activities on a sunny day. | 5 (1.30) | 14 (3.63) | 44 (11.40) | 195 (50.52) | | 128 (33.16) |
| 31 | Sunscreen should be applied when going swimming at the pool, beach, or waterfall. | 3 (0.78) | 24 (6.22) | 51 (13.21) | 179 (46.37) | | 129 (33.42) |
| 32 | Sunscreen should be applied when attending any occasion at night. | 40 (10.36) | 125 (32.38) | 88 (22.80) | 107 (27.72) | | 26 (6.74) |
| 33 | Avoiding excessive exposure to sunlight, wearing sunscreen when going out, and covering most body parts are the best preventive measures. | 9 (2.33) | 18 (4.66) | 49 (12.69) | 202 (52.33) | | 108 (27.98) |
| 34 | Sunbathing is good for my skin. | 26 (6.74) | 85 (22.02) | 136 (35.23) | 132 (34.20) | | 7 (1.81) |
| 35 | I eat antioxidant-rich foods such as carrots and tomatoes to prevent skin cancer. | 11 (2.85) | 28 (7.25) | 91 (23.58) | 206 (53.37) | | 50 (12.95) |

**Table S3 Practice of participants towards skin cancer**

| **No.** | **Statement** | **Never** | **Rarely** | **Sometimes** | **Often** | **Always** |
| --- | --- | --- | --- | --- | --- | --- |
| 1 | I examine my skin (head to toe) thoroughly annually. | 116 (30.05) | 90  (23.32) | 78  (20.21) | 90  (23.32) | 12  (3.11) |
| 2 | I see a skin specialist at least once a year for a professional skin exam. | 188  (48.70) | 61  (15.80) | 52  (13.47) | 74  (19.17) | 11  (2.85) |
| 3 | I do the skin biopsy test every 2 years. | 203  (52.59) | 48  (12.44) | 63  (16.32) | 58  (15.03) | 14  (3.63) |
| 4 | I do the skin exam with health care professionals to see whether there are abnormalities in my skin (colour, shape, texture). | 183  (47.41) | 55  (14.25) | 71  (18.39) | 64  (16.58) | 13  (3.37) |
| 5 | I ignore the worrisome change. | 60  (15.54) | 82  (21.24) | 118  (30.57) | 88  (22.80) | 38  (9.84) |
| 6 | I will self-examine the skin and check for abnormal signs. | 61  (15.80) | 83  (21.50) | 125  (32.38) | 100  (25.91) | 17  (4.40) |
| 7 | I wear the hat with a wide brim (all around) to shade my face, head, ears, and neck when I go outside. | 98  (25.39) | 103  (26.68) | 90  (23.32) | 73  (18.91) | 22  (5.70) |
| 8 | I stay under a patio umbrella which is made up of sunbrella when having tea with friends outside the cafe. | 30  (7.77) | 34  (8.81) | 94  (24.35) | 154  (39.90) | 74  (19.17) |
| 9 | I apply sunscreen 30 minutes before I plan to go out and when outdoors, reapply sunscreen every 2 hours. | 84  (21.76) | 95  (24.61) | 102  (26.42) | 87  (22.54) | 18  (4.66) |
| 10 | I do not reapply sunscreen after excessive swimming or excessive sweating. | 74  (19.17) | 79  (20.47) | 89  (23.06) | 92  (23.83) | 52  (13.47) |
| 11 | I use a stronger (UVA/UVB) sunscreen with a sun protection factor (SPF 50) or higher. | 56  (14.51) | 61  (15.80) | 111  (28.76) | 93  (24.09) | 65  (16.84) |
| 12 | I just apply sunscreen without checking its expiry date. | 126  (32.64) | 86  (22.28) | 67  (17.36) | 80  (20.73) | 27  (6.99) |
| 13 | I avoid sunburn by applying sunscreen to reduce the risk of skin cancer. | 42  (10.88) | 40  (10.36) | 98  (25.39) | 148  (38.34) | 58  (15.03) |
| 14 | I use sunscreen in the form of lotion instead of spray. | 41  (10.62) | 38  (9.84) | 87  (22.54) | 108  (27.98) | 112  (29.02) |
| 15 | I apply a two-finger rule which is by squeezing a small amount to the palm and spread to the tip of the index and middle fingers when applying sunscreen for the full body. | 83  (21.50) | 73  (18.91) | 114  (29.53) | 83  (21.50) | 33  (8.55) |
| 16 | I wear singlets when going outside. | 152  (39.38) | 94  (24.35) | 77  (19.95) | 55  (14.25) | 8  (2.07) |
| 17 | I wear UV protective clothing when I go outside. | 141  (36.53) | 71  (18.39) | 82  (21.24) | 80  (20.73) | 12  (3.11) |
| 18 | I wear clothing made up of fabric (polyester, nylon, wool) to prevent UV rays. | 119  (30.83) | 101  (26.17) | 93  (24.09) | 59  (15.28) | 14  (3.63) |
| 19 | I wear more layers of clothing to prevent UV rays when I go hiking and jogging at parks and beaches. | 119  (30.83) | 104  (26.94) | 79  (20.47) | 68  (17.62) | 16  (4.15) |
| 20 | I wear bright-coloured clothing when I go outside. | 33  (8.55) | 96  (24.87) | 153  (39.64) | 86  (22.28) | 18  (4.66) |
| 21 | I like to swim during the noon time. | 156  (40.41) | 92  (23.83) | 73  (18.91) | 50  (12.95) | 15  (3.89) |
| 22 | I reapply sunscreen every 40-80 minutes when swimming. | 173  (44.82) | 73  (18.91) | 68  (17.62) | 61  (15.80) | 11  (2.85) |
| 23 | I do not reapply sunscreen after towelling. | 94  (24.35) | 57  (14.77) | 89  (23.06) | 82  (21.24) | 64  (16.58) |
| 24 | I wear a long swimming suit when swimming. | 152  (39.38) | 75  (19.43) | 73  (18.91) | 65  (16.84) | 21  (5.44) |
| 25 | For water resistant activity (swimming), I apply (UVA/UVB) sunscreen with an SPF of 50 or higher. | 91  (23.58) | 50  (12.95) | 92  (23.83) | 102  (26.42) | 51  (13.21) |
| 26 | I spend most of my time playing with kids outside like in the garden or playground for an hour or two. | 106  (27.46) | 101  (26.17) | 100  (25.91) | 75  (19.43) | 4  (1.04) |
| 27 | I do not choose my work area next to the window in the office. | 56  (14.51) | 79  (20.47) | 111  (28.76) | 96  (24.87) | 44  (11.40) |
| 28 | I help remove any debris at the construction site for 1-2 hours without using any sun protection things. | 145  (37.56) | 90  (23.32) | 63  (16.32) | 72  (18.65) | 16  (4.15) |
| 29 | I apply sunscreen before planting and harvesting the crops for 1-2 hours every day. | 126  (32.64) | 56  (14.51) | 67  (17.36) | 91  (23.58) | 46  (11.92) |
