# Supplementary material for: Knowledge, attitudes and practices toward skin cancer prevention among Malaysian adults: a cross-sectional online survey
Source: BMJ Open. 2026 Feb 22;16(2):e103040. doi: 10.1136/bmjopen-2025-103040 (PMC12927299; doi:10.1136/bmjopen-2025-103040)
Supplement: online supplemental file 4 [file bmjopen-16-2-s004.pdf]

Table 1 Sociodemographic characteristics of participants

Variables	Frequency	Percentage (%)
**Age**	27.84 ± 16.9	
Young adult (18-35)	333	86.27
Middle adult (36-55)	44	11.40
Old adult (>55)	9	2.33
**Gender**		
Male	214	55.44
Female	172	44.56
**Ethnicity**		
Malay	97	25.13
Chinese	253	65.54
Indian	13	3.37
Others	23	5.96
**Level of education**		
Primary	1	0.26
Secondary	88	22.80
Tertiary	297	76.94
**Living area**		
Urban	334	86.53
Rural	52	13.47
**When do you mainly stay outdoors?**		
Early morning before 11am and/or late afternoon/evening (after 3pm)	159	41.19
Midday (11am-3pm)	119	30.83
All day (Sunrise to sunset)	69	17.88

Not applicable	39	10.10
**When going outdoors, how long are you exposed to the sun?**		
Less than one hour	190	49.22
Between 1-3 hours per day	148	38.34
Greater than 3 hours per day	43	11.14
Not applicable	5	1.30
**Do you have a personal history of skin cancer?**		
Yes	92	23.83
No	294	76.17
**Do you have a family history of skin cancer?**		
Yes	66	17.10
No	320	82.90
**Do you have a diagnosis of skin disease? (e.g., eczema, acne, psoriasis, dermatitis)**		
Yes	179	46.37
No	207	53.63
**What is your skin tone?**		
Light	15	3.89
Fair	158	40.93
Tan	190	49.22
Deep	23	5.96
**What is your skin type?**		
Type 1: skin always burns, never tans, and is sensitive to UV exposure	17	4.40
Type 2: skin burns easily and tans minimally	65	16.84
Type 3: skin burns moderately and tans gradually to light brown	90	23.32

Type 4: skin burns minimally and always tans well to moderately brown	85	22.02
Type 5: skin rarely burns and tans profusely to dark	88	22.80
Type 6: skin never burns, is deeply pigmented, and is least sensitive to UV exposure	41	10.62
**How many moles do you have on the skin?**		
Low (0-50)	319	82.64
Moderate (51-100)	61	15.80
High (>100)	6	1.55
**Do you have unusual moles (asymmetrical, irregular shape/border, uneven colour, diameter >6mm, growing moles)? ABCDE rule**		
Yes	91	23.58
No	288	74.61
Not applicable	7	1.81
**Do you have any problem with hair loss or thinning?**		
Yes	191	49.48
No	195	50.52
**Does your skin freckle?**		
Yes	150	38.86
No	236	61.14
**Do you ever have a severe sunburn that blisters?**		
Yes	118	30.57
No	268	69.43

Table 2 Statistical differences between sociodemographic with the participant's knowledge, attitude and practice about skin cancer among Malaysian population

		Knowledge		Attitude		Practice	
**Sociodemographic data**		**Knowledge scores mean**	**P-value**	**Attitude scores mean**	**P-value**	**Practice scores mean**	**P-value**
Gender	Male	13.67	0.320	18.11	**0.002**	10.85	0.906
	Female	14.24		20.23		10.80	
Ethnicity	Malay	15.48	**0.004**	20.64	**0.001**	13.21	**0.001**
	Chinese	13.47		19.11		9.87	
	Indian	10.92		14.77		9.46	
	Others	14.00		14.17		12.09	
Level of education	Primary (e.g., UPSR)	10.00	**0.032**	10.00	**0.001**	16.00	0.161
	Secondary (e.g., PMR, PT3, SPM, STPM, O-	12.61		15.95		11.44	

	Levels)						
	Tertiary (e.g., Pre-University, Diploma, Bachelor, Master, Ph.D.)	14.32		20.01		10.63	
Living area	Urban	13.76	0.158	18.96	0.484	10.68	0.107
	Rural	14.94		19.65		11.75	
When do you mainly stay outdoors	Early morning before 11am and/or late afternoon/evening (after 3pm)	14.36	0.124	19.40	0.130	10.97	0.073
	Midday (11am-3pm)	13.73		18.69		11.45	

	All day (Sunrise to sunset)	12.68		17.91		9.83	
	Not applicable	14.92		20.82		10.13	
When going outdoors, how long are you exposed to the sun?	Less than one hour	14.22	0.641	20.05	**0.034**	10.22	**0.011**
	Between 1-3 hours per day	13.55		17.97		11.74	
	Greater than 3 hours per day	14.07		18.44		10.58	
	Not applicable	12.20		18.80		8.80	
Do you have a personal history of	Yes	13.43	0.339	16.89	**0.001**	13.14	**0.001**
	No	14.07		19.73		10.10	

skin cancer?							
Do you have a family history of skin cancer?	Yes	14.09	0.788	16.85	**0.003**	13.65	**0.001**
	No	13.89		19.51		10.24	
Do you have a diagnosis of skin disease? (e.g., eczema, acne, psoriasis, dermatitis)	Yes	14.95	**0.001**	20.03	**0.007**	12.10	**0.001**
	No	13.03		18.21		9.72	
What is your skin tone?	Light	10.87	0.113	13.73	**0.006**	8.60	0.244
	Fair	14.25		18.72		11.02	
	Tan	13.76		19.75		10.87	

	Deep	14.96		19.09		10.57	
What is your skin type?	Type 1: skin always burns, never tans, and is sensitive to UV exposure	12.88	0.566	17.65	0.152	9.94	**0.007**
	Type 2: skin burns easily and tans minimally	13.74		17.85		11.55	
	Type 3: skin burns moderately and tans gradually to light brown	13.82		18.93		11.62	
	Type 4:	14.91		20.61		11.35	

	skin burns minimally and always tans well to moderately brown						
	Type 5: skin rarely burns and tans profusely to dark	13.67		19.14		9.85	
	Type 6: skin never burns, is deeply pigmented, and is least sensitive to UV exposure	13.37		18.44		9.29	

How many moles do you have on the skin?	Low (0-50)	13.90	0.542	19.12	0.626	10.46	**0.001**
	Moderate (51-100)	14.28		18.97		12.74	
	High (>100)	11.67		16.50		10.83	
Do you have unusual moles (asymmetrical, irregular shape/border, uneven colour, diameter > 6mm, growing moles)? ABCDE rule	Yes	14.36	0.170	18.43	0.227	12.76	**0.001**
	No	13.87		19.33		10.25	
	Not applicable	10.29		15.86		9.29	

Do you have any problem with hair loss or thinning?	Yes	13.66	0.362	18.52	0.113	11.23	0.081
	No	14.18		19.58		10.44	
Does your skin freckle?	Yes	13.55	0.302	17.97	**0.010**	11.71	**0.002**
	No	14.16		19.75		10.27	
Do you ever have a severe sunburn that blisters?	Yes	13.58	0.421	17.48	**0.002**	12.33	**0.001**
	No	14.07		19.75		10.16	

Table 3 Significant relationships between the levels of knowledge and demographic variables

**Variables**	**Respondents (%)**			
	**Poor**	**Moderate**	**Good**	**$\chi2$; p-value**

Gender	Male	122 (57.0%)	84 (39.3%)	8 (3.7%)	3.096; 0.213
	Female	94 (54.7%)	76 (44.2%)	2 (1.2%)	
Ethnicity	Malay	41 (42.3%)	49 (50.5%)	7 (7.2%)	23.657; **0.001**
	Chinese	148 (58.5%)	102 (40.3%)	3 (1.2%)	
	Indian	12 (92.3%)	1 (7.7%)	0 (0.0%)	
	Others	15 (65.2%)	8 (34.8%)	0 (0.0%)	
Level of education	Primary (eg. UPSR)	1 (100.0%)	0 (0.0%)	0 (0.0%)	8.941; 0.063
	Secondary (eg. PMR, PT3, SPM, STPM, O-Levels)	60 (68.2%)	25 (28.4%)	3 (3.4%)	
	Tertiary (eg. Pre-University, Diploma, Bachelor, Master, Ph.D.)	155 (52.2%)	135 (45.5%)	7 (2.4%)	
Living area	Urban	191 (57.2%)	133 (39.8%)	10 (3.0%)	3.814; 0.149
	Rural	25 (48.1%)	27 (51.9%)	0 (0.0%)	

When do you mainly stay outdoors	Early morning before 11am and/or late afternoon/evening (after 3pm)	82 (48.5%)	80 (47.3%)	7 (4.1%)	9.579; 0.144
	Midday (11am-3pm)	67 (56.3%)	52 (43.7%)	0 (0.0%)	
	All day (Sunrise to sunset)	46 (66.7%)	21 (30.4%)	2 (2.9%)	
	Not applicable	21 (53.8%)	17 (43.6%)	1 (2.6%)	
When going outdoors, how long are you exposed to the sun?	Less than one hour	102 (53.7%)	82 (43.2%)	6 (3.2%)	3.053; 0.802
	Between 1-3 hours per day	85 (57.4%)	59 (39.9%)	4 (2.7%)	
	Greater than 3 hours per day	25 (58.1%)	18 (41.9%)	0 (0.0%)	
	Not applicable	4 (80.0%)	1 (20.0%)	0 (0.0%)	

Do you have a personal history of skin cancer?	Yes	62 (67.4%)	28 (30.4%)	2 (2.2%)	6.439; **0.040**
	No	154 (52.4%)	132 (44.9%)	8 (2.7%)	
Do you have a family history of skin cancer?	Yes	40 (60.6%)	22 (33.3%)	4 (6.1%)	5.273; 0.072
	No	176 (55.0%)	138 (43.1%)	6 (1.9%)	
Do you have a diagnosis of skin disease? (e.g., eczema, acne, psoriasis, dermatitis)	Yes	86 (48.0%)	87 (48.6%)	6 (3.4%)	8.602; **0.014**
	No	130 (62.8%)	73 (35.3%)	4 (1.9%)	
What is your skin tone?	Light	14 (93.3%)	1 (6.7%)	0 (0.0%)	13.514; **0.036**
	Fair	83 (52.5%)	70 (44.3%)	5 (3.2%)	
	Tan	110 (57.9%)	75 (39.5%)	5 (2.6%)	

	Deep	9 (39.1%)	14 (60.9%)	0 (0.0%)	
What is your skin type?	Type 1: skin always burns, never tans, and is sensitive to UV exposure	11 (64.7%)	6 (35.3%)	0 (0.0%)	6.027; 0.813
	Type 2: skin burns easily and tans minimally	37 (56.9%)	25 (38.5%)	3 (4.6%)	
	Type 3: skin burns moderately and tans gradually to light brown	53 (58.9%)	36 (40.0%)	1 (1.1%)	
	Type 4: skin burns minimally and always tans well to moderately brown	44 (51.8%)	37 (43.5%)	4 (4.7%)	
	Type 5: skin	48 (54.5%)	38 (43.2%)	2 (2.3%)	

	rarely burns and tans profusely to dark				
	Type 6: skin never burns, is deeply pigmented, and is least sensitive to UV exposure	23 (56.1%)	18 (43.9%)	0 (0.0%)	
How many moles do you have on the skin?	Low (0-50)	177 (55.5%)	133 (41.7%)	9 (2.8%)	2.165; 0.706
	Moderate (51-100)	34 (55.7%)	26 (42.6%)	1 (1.6%)	
	High (>100)	5 (83.3%)	1 (16.7%)	0 (0.0%)	
Do you have unusual moles (asymmetrical, irregular shape/border, uneven colour,	Yes	53 (58.2%)	35 (38.5%)	3 (3.3%)	3.306; 0.508
	No	157 (54.5%)	124 (43.1%)	7 (2.4%)	
	Not applicable	6 (85.7%)	1 (14.3%)	0 (0.0%)	

diameter >6 mm, growing moles)? ABCDE rule					
Do you have any problem with hair loss or thinning?	Yes	111 (58.1%)	78 (40.8%)	2 (1.0%)	3.826; 0.148
	No	105 (53.8%)	82 (42.1%)	8 (4.1%)	
Does your skin freckle?	Yes	91 (60.7%)	56 (37.3%)	3 (2.0%)	2.306; 0.316
	No	125 (53.0%)	104 (44.1%)	7 (3.0%)	
Do you ever have a severe sunburn that blisters?	Yes	67 (56.8%)	48 (40.7%)	3 (2.5%)	0.046; 0.977
	No	149 (55.6%)	112 (41.8%)	7 (2.6%)	

Table 4 Significant relationships between the levels of attitude and demographic variables

**Variables**	**Respondents (%)**			
	**Poor**	**Moderate**	**Good**	**$\chi2$; p-value**

Gender	Male	91 (42.5%)	112 (52.3%)	11 (5.1%)	11.815; **0.003**
	Female	45 (26.2%)	119 (69.2%)	8 (4.7%)	
Ethnicity	Malay	26 (26.8%)	65 (67.0%)	6 (6.2%)	32.774; **0.001**
	Chinese	82 (32.4%)	158 (62.5%)	13 (5.1%)	
	Indian	10 (76.9%)	3 (23.1%)	0 (0.0%)	
	Others	18 (78.3%)	5 (21.7%)	0 (0.0%)	
Level of education	Primary (eg. UPSR)	1 (100.0%)	0 (0.0%)	0 (0.0%)	36.528; **0.001**
	Secondary (eg. PMR, PT3, SPM, STPM, O-Levels)	54 (61.4%)	32 (36.4%)	2 (2.3%)	
	Tertiary (eg. Pre-University, Diploma, Bachelor, Master,	81 (27.3%)	199 (67.0%)	17 (5.7%)	

	Ph.D.)				
Living area	Urban	120 (35.9%)	196 (58.7%)	18 (5.4%)	1.997; 0.368
	Rural	16 (30.8%)	35 (67.3%)	1 (1.9%)	
When do you mainly stay outdoors	Early morning before 11am and/or late afternoon/evening (after 3pm)	49 (30.8%)	100 (62.9%)	10 (6.3%)	13.072; **0.042**
	Midday (11am-3pm)	47 (39.5%)	69 (58.0%)	3 (2.5%)	
	All day (Sunrise to sunset)	30 (43.5%)	38 (55.1%)	1 (1.4%)	
	Not applicable	10 (25.6%)	24 (61.5%)	5 (12.8%)	
When going outdoors,	Less than one hour	54 (28.4%)	124 (65.3%)	12 (6.3%)	10.118; 0.120

how long are you exposed to the sun?	Between 1-3 hours per day	64 (43.2%)	78 (52.7%)	6 (4.1%)	
	Greater than 3 hours per day	17 (39.5%)	25 (58.1%)	1 (2.3%)	
	Not applicable	1 (20.0%)	4 (80.0%)	0 (0.0%)	
Do you have a personal history of skin cancer?	Yes	48 (52.7%)	41 (45.1%)	2 (2.2%)	17.708; **0.001**
	No	87 (29.6%)	190 (64.6%)	17 (5.8%)	
Do you have a family history of skin cancer?	Yes	36 (54.5%)	25 (37.9%)	5 (7.6%)	15.985; **0.001**
	No	100 (31.3%)	206 (64.4%)	14 (4.4%)	
Do you have a diagnosis of skin	Yes	55 (30.7%)	112 (62.6%)	12 (6.7%)	4.491; 0.106
	No	81 (39.1%)	119 (57.5%)	7 (3.4%)	

disease? (e.g., eczema, acne, psoriasis, dermatitis)					
What is your skin tone?	Light	11 (73.3%)	3 (20.0%)	1 (6.7%)	13.681; **0.033**
	Fair	59 (37.3%)	91 (57.6%)	8 (5.1%)	
	Tan	58 (32.2%)	112 (62.2%)	10 (5.6%)	
	Deep	8 (34.8%)	15 (65.2%)	0 (0.0%)	
What is your skin type?	Type 1: skin always burns, never tans, and is sensitive to UV exposure	8 (47.1%)	8 (47.1%)	1 (5.9%)	7.462; 0.681
	Type 2: skin burns easily and tans minimally	28 (43.1%)	34 (52.3%)	3 (4.6%)	

	Type 3: skin burns moderately and tans gradually to light brown	30 (33.3%)	57 (63.3%)	3 (3.3%)	
	Type 4: skin burns minimally and always tans well to moderately brown	24 (28.2%)	56 (65.9%)	5 (5.9%)	
	Type 5: skin rarely burns and tans profusely to dark	33 (37.5%)	49 (55.7%)	6 (6.8%)	
	Type 6: skin never burns, is deeply	13 (31.7%)	27 (65.9%)	1 (2.4%)	

	pigmented, and is least sensitive to UV exposure				
How many moles do you have on the skin?	Low (0-50)	109 (34.2%)	193 (60.5%)	17 (5.3%)	3.363; 0.499
	Moderate (51-100)	23 (37.7%)	36 (59.0%)	2 (3.3%)	
	High (>100)	4 (66.7%)	2 (33.3%)	0 (0.0%)	
Do you have unusual moles (asymmetrical, irregular shape/border, uneven colour, diameter >6 mm, growing moles)? ABCDE rule	Yes	38 (41.8%)	50 (54.9%)	3 (3.3%)	3.195; 0.526
	No	95 (33.0%)	177 (61.5%)	16 (5.6%)	
	Not applicable	3 (42.9%)	4 (57.1%)	0 (0.0%)	
Do you have	Yes	76 (39.8%)	108 (56.5%)	7 (3.7%)	4.131; 0.127

any problem with hair loss or thinning?	No	60 (30.8%)	123 (63.1%)	12 (6.2%)	
Does your skin freckle?	Yes	69 (46.0%)	76 (50.7%)	5 (3.3%)	12.784; **0.002**
	No	67 (28.4%)	155 (65.7%)	14 (5.9%)	
Do you ever have a severe sunburn that blisters?	Yes	57 (48.3%)	58 (49.2%)	3 (2.5%)	13.445; **0.001**
	No	79 (29.5%)	173 (64.6%)	16 (6.0%)	

Table 5 Significant relationships between the levels of practice and demographic variables

**Variables**		**Respondents (%)**			
		**Poor**	**Moderate**	**Good**	**χ2; p-value**
Gender	Male	179 (83.6%)	32 (15.0%)	3 (1.4%)	0.648; 0.723
	Female	146 (84.9%)	25 (14.5%)	1 (0.6%)	
Ethnicity	Malay	59 (60.8%)	34 (35.1%)	4 (4.1%)	60.210; **0.001**
	Chinese	236 (93.3%)	17 (6.7%)	0 (0.0%)	
	Indian	12 (92.3%)	1 (7.7%)	0 (0.0%)	

	Others	18 (78.3%)	5 (21.7%)	0 (0.0%)	
Level of education	Primary (eg. UPSR)	0 (0.0%)	1 (100.0%)	0 (0.0%)	14.422; **0.006**
	Secondary (eg. PMR, PT3, SPM, STPM, O-Levels)	68 (77.3%)	17 (19.3%)	3 (3.4%)	
	Tertiary (eg. Pre-University, Diploma, Bachelor, Master, Ph.D)	257 (86.5%)	39 (13.1%)	1 (0.3%)	
Living area	Urban	285 (85.3%)	47 (14.1%)	2 (0.6%)	5.767; 0.056
	Rural	40 (76.9%)	10 (19.2%)	2 (3.8%)	
When do you mainly stay outdoors	Early morning before 11am	130 (81.8%)	27 (17.0%)	2 (1.3%)	10.577; 0.102

	and/or late afternoon/evening (after 3pm)				
	Midday (11am-3pm)	94 (79.0%)	23 (19.3%)	2 (1.7%)	
	All day (Sunrise to sunset)	65 (94.2%)	4 (5.8%)	0 (0.0%)	
	Not applicable	36 (92.3%)	3 (7.7%)	0 (0.0%)	
When going outdoors, how long are you exposed to the sun?	Less than one hour	171 (90.0%)	18 (9.5%)	1 (0.5%)	16.240; **0.013**
	Between 1-3 hours per day	111 (75.0%)	34 (23.0%)	3 (2.0%)	
	Greater than 3 hours per day	38 (88.4%)	5 (11.6%)	0 (0.0%)	

	Not applicable	5 (100.0%)	0 (0.0%)	0 (0.0%)	
Do you have a personal history of skin cancer?	Yes	60 (65.2%)	30 (32.6%)	2 (2.2%)	32.715; **0.001**
	No	265 (90.1%)	27 (9.2%)	2 (0.7%)	
Do you have a family history of skin cancer?	Yes	40 (60.6%)	23 (34.8%)	3 (4.5%)	36.465; **0.001**
	No	285 (89.1%)	34 (10.6%)	1 (0.3%)	
Do you have a diagnosis of skin disease? (e.g., eczema, acne, psoriasis, dermatitis)	Yes	133 (74.3%)	43 (24.0%)	3 (1.7%)	24.563; **0.001**
	No	192 (92.8%)	14 (6.8%)	1 (0.5%)	
What is your	Light	15 (100.0%)	0 (0.0%)	0 (0.0%)	3.313; 0.769

skin tone?	Fair	132 (83.5%)	24 (15.2%)	2 (1.3%)	
	Tan	159 (83.7%)	29 (15.3%)	2 (1.1%)	
	Deep	19 (82.6%)	4 (17.4%)	0 (0.0%)	
What is your skin type?	Type 1: skin always burns, never tans, and is sensitive to UV exposure	16 (94.1%)	1 (5.9%)	0 (0.0%)	24.371; **0.007**
	Type 2: skin burns easily and tans minimally	50 (76.9%)	13 (20.0%)	2 (3.1%)	
	Type 3: skin burns moderately and tans gradually to light brown	67 (74.4%)	23 (25.6%)	0 (0.0%)	
	Type 4: skin	72 (84.7%)	11 (12.9%)	2 (2.4%)	

	burns minimally and always tans well to moderately brown				
	Type 5: skin rarely burns and tans profusely to dark	83 (94.3%)	5 (5.7%)	0 (0.0%)	
	Type 6: skin never burns, is deeply pigmented, and is least sensitive to UV exposure	37 (90.2%)	4 (9.8%)	0 (0.0%)	
How many moles do you have on the	Low (0-50)	279 (87.5%)	36 (11.3%)	4 (1.3%)	23.404; **0.001**
	Moderate (51-100)	40 (65.6%)	21 (34.4%)	0 (0.0%)	

skin?	High (>100)	6 (100.0%)	0 (0.0%)	0 (0.0%)	
Do you have unusual moles (asymmetrical, irregular shape/border, uneven colour, diameter >6 mm, growing moles)? ABCDE rule	Yes	63 (69.2%)	27 (29.7%)	1 (1.1%)	21.762; **0.001**
	No	255 (88.5%)	30 (10.4%)	3 (1.0%)	
	Not applicable	7 (100.0%)	0 (0.0%)	0 (0.0%)	
Do you have any problem with hair loss or thinning?	Yes	152 (79.6%)	38 (19.9%)	1 (0.5%)	8.650; **0.013**
	No	173 (88.7%)	19 (9.7%)	3 (1.5%)	
Does your skin freckle?	Yes	116 (77.3%)	32 (21.3%)	2 (1.3%)	8.745; **0.013**
	No	209 (88.6%)	25 (10.6%)	2 (0.8%)	
Do you ever	Yes	84 (71.2%)	33 (28.0%)	1 (0.8%)	23.527;

have a severe sunburn that blisters?	No	241 (89.9%)	24 (9.0%)	3 (1.1%)	**0.001**

Table 6. Factors associated with poor skin cancer preventive practices among Malaysian adults (n = 386)

Variable	Adjusted OR	95% CI	p-value
Male sex	1.85	1.22–2.80	0.004
Age ≥30 years	1.31	0.88–1.96	0.182
Non-tertiary education	2.14	1.39–3.30	0.001
Urban residence	0.92	0.58–1.46	0.732
Light skin tone	1.76	1.05–2.94	0.032
Personal history of skin cancer	0.58	0.36–0.93	0.024
History of severe sunburn	1.67	1.10–2.54	0.016

Notes: Odds ratios (ORs) were obtained using multivariable logistic regression. Poor preventive practice was coded as the outcome (1 = poor; 0 = moderate/good). Confidence intervals (CI) are 95%.
