# Supplementary material for: Knowledge, attitudes and practices toward skin cancer prevention among Malaysian adults: a cross-sectional online survey
Source: BMJ Open. 2026 Feb 22;16(2):e103040. doi: 10.1136/bmjopen-2025-103040 (PMC12927299; doi:10.1136/bmjopen-2025-103040)
Supplement: Supplementary file 5 [file bmjopen-2025-103040.reviewer_comments.pdf]

PEER REVIEW HISTORY

BMJ Open publishes all reviews undertaken for accepted manuscripts. Reviewers are asked to complete a checklist review form and are provided with free text boxes to elaborate on their assessment. These free text comments are reproduced below.

ARTICLE DETAILS

Title (Provisional)

Knowledge, Attitudes and Practices Toward Skin Cancer Prevention Among Malaysian Adults: A Cross-Sectional Online Survey

Authors

Mohammed, Ali Haider; Hassan, Bassam Abdul Rasool; Wong, Yen Jun; Ying, Loh Hui; Hong, Marcus Loh Boon; Nee, Annabel Wong Sze; Ying, Lo Siew; Ramachandram, Dinesh Sangarran; Hassan, Hawar Sardar; Jia, Lee Jia; Dujaili, Juman; Blebil, Ali

---

VERSION 1 - REVIEW

**Reviewer**	**1**
**Name**	**Dewi, Ayu Putu Madri**
**Affiliation**	**Amsterdam UMC Location AMC, Epidemiology and Data Science**
**Date**	**14-Apr-2025**
**COI**	**None**

Manuscript Title: Skin Cancer Prevention: Bridging the Gap in Knowledge, Attitude, and Practice in Malaysia

Dear authors,

I am an Open Science researcher, who has checked your manuscript for its adherence to a number of Open Science practices.

Below you find the checklist that I used to assess your manuscript for adherence to a number of these practices.

Based on this assessment, I would suggest the following refinements/additions:

1. Availability of a protocol: Please provide a (link to the) the protocol.

2. Pre-registration: Please provide information on whether and where the study was pre-registered.

3. Open Materials: OK

4. Open Data: Given the confidentiality of the data, please specify whether de-identified, metadata, or a subset of the data can still be made publicly available.

Note, if this paper is accepted, you will be required to add a data availability statement and may be required to share data by BMJ/BMJ Open Policy. For more information, please see: https://authors.bmj.com/policies/data-sharing/ ; https://bmjopen.bmj.com/pages/authors

5. Open Code: Please provide (a link to) the code used for the analyses in this study.

Note that for papers accepted at The BMJ you will be required to provide a supplementary file with the code if this paper is accepted. For more information, please see Data sharing section on the website: https://authors.bmj.com/policies/data-sharing/ (The BMJ); https://bmjopen.bmj.com/pages/authors (BMJ Open)

6. Citations: Please provide citations for your materials/data/codes.

7. Software and tools used are described: OK

8. Open access publication: OK

9. Reporting checklist provided: OK

10. Preprint: Please state explicitly whether and where the preprint of this study can be found.

Reviewer 2

Name Khamees, Almu'atasim

Affiliation Hashemite Kingdom of Jordan Ministry of Health, Department of General Surgery

Date 17-Apr-2025

COI None

Dear Authors,

The objective of the study is valuable. The topic is very interesting. The study assesses the KAP (Knowledge, Attitude, Practice) regarding skin cancer among Malaysian adults. They found significant gaps in knowledge and practice despite relatively positive attitudes. However, some points need to be addressed, as mentioned below:

Title:

Good and meaningful.

Abstract:

The abstract explains the contents of the manuscript.

Introduction:

Good

Methods:

Appropriate detailed methodology and statistical analysis.

Results:

How did your participants have a positive attitude toward skin cancer while moderate in Figure 1? (page 11, line 35)

Please be specific in the description. However, the other descriptions of the results were good.

Discussion:

As you have great results, the discussion should focus more on comparisons with similar studies.

Also, there is an Insufficient Discussion of Cultural Factors.

As there is a big gap in KAP, as presented in the manuscript, you should expand on the implications of the findings for policy-making and public health campaigns in Malaysia.

While the exclusion of Healthcare Professionals was intended to reduce bias, this exclusion limits understanding of how healthcare workers' KAP might differ from the general population, which could inform training programs. You can discuss this in the discussion and highlight the importance of the healthcare level of education. It would be helpful if you read and cite the following items: PMID: 35198247 and 31431832 or similar articles.

Conclusion:

Good

References:

References are comprehensive, but more references are needed for the discussion section.

Tables and Figures:

Tables and figures enhance data interpretation.

Limitations:

A lot of young and urban population.

Predominantly, Chinese participants (65.54%) may not accurately reflect Malaysia's multi-ethnic population.

**Reviewer**	**3**
**Name**	**Allela, Omer**
**Affiliation**	**Al-Noor University College**

**Date**	**08-Oct-2025**
**COI**	**None**

Important Suggestions

1. Clearly state the goals of the study

The objectives are expressed in broad strokes. The article would be strengthened if it included quantifiable goals like:

"To ascertain how sociodemographic characteristics and KAP levels relate to Malaysians' prevention of skin cancer."

"To find predictors of effective preventive measures."

2. Generalisability and Sampling

Social media snowball sampling creates selection bias by probably over-representing urban and educated populations.

Suggested:

Clearly state this restriction in the discussion.

To increase representativeness, recommend stratified techniques or random sampling in the future.

3. Data Reliability and Validity

Although the KAP-SC-Q is cited as "validated" in the paper, the validation specifics (such as Cronbach's α and factor structure) are only addressed in passing and are based on a prior publication.

For transparency, propose adding a summary table of the most important psychometric indices.

4. Reporting on Statistics

Inconsistently reported p-values or percentages without confidence intervals can be seen in the results sections.

Give precise p-values and 95% CIs for BMJ Open standards.

To demonstrate the strength of relationships, logistic regression findings should offer odds ratios (OR) with confidence intervals (CIs).

5. Depth of Discussion

More direct connections between the findings and current regional or international KAP investigations should be made in the discussion.

Compare with comparable research conducted in Southeast Asia, such as Thailand, Indonesia, and Singapore.

Talk about any behavioural or cultural obstacles that might be influencing preventative strategies.

6. Section on Limitations

Extend to incorporate:

Online recruiting and an over-representation of Chinese and urban participants are examples of sampling bias.

risk of social desirability bias in self-reported data.

Cross-sectional design prevents causal inference.

7. Formatting and Language

Although there are a few small grammatical and stylistic errors, the work is generally well written.

For instance, "with the intention of creating an instrument tool" → "with the goal of creating an instrument to evaluate..."

"...poor skin cancer prevention practices (84.2%)" ← "...84.2% showed inadequate preventive measures."

Make sure the tense is constant (use the past tense for techniques and results).

VERSION 1 - AUTHOR RESPONSE

Response to Reviewer 1

Comment : Availability of a protocol
"Please provide a (link to) the protocol."
Response:
The ethics application (MUHREC-36701) served as the study protocol. A separate pre-registered protocol was not developed. This clarification has been added in the Ethics Approval section.

Comment : Pre-registration
"Please provide information on whether and where the study was pre-registered."
Response:
The study was **not preregistered**, as it was observational and exploratory in nature.

Comment : Open Data
"Please specify whether de-identified data can be made publicly available."
Response:
A Data/Code Availability Statement has now been added indicating that de-identified data, the coded dataset, and the survey instrument are available in a public repository (https://drive.google.com/drive/folders/1a5-irJmoRNmcW0oxnO4K-

jjoXunpDcsg?usp=drive_link). All data comply with MUHREC de-identification requirements.

Comment : Open Code
"Please provide (a link to) the code used for the analyses in this study."
Response:
The SPSS syntax file used for analysis has been uploaded and is accessible in the same public repository linked in the Data/Code Availability Statement.

Comment : Citations for materials/data/code
Response:
A citation to the data repository link has been added in the Data/Code Availability Statement.

Comment : Preprint disclosure
"Please state explicitly whether and where the preprint of this study can be found."
Response:
No preprint has been submitted for this study. This statement has been added to the Data/Code Availability section.

Response to Reviewer 2

Comment: Issue of positive attitude vs. moderate classification in Figure 1.
"How did your participants have a positive attitude toward skin cancer while moderate in Figure 1? Please be specific in the description."
Response:
Thank you for this observation. The result description has been clarified. Although the mean attitude score falls within the moderate range, most individual attitude items received positive responses (agree/strongly agree), which we now explicitly state. This clarification has been added in the Results section.

Comment: Discussion requires more comparison with similar studies.
Response:
Additional comparisons have been incorporated in the Discussion, including regional evidence from Jordan and Saudi Arabia, showing similar gaps between awareness and preventive practices (PMID: 35198247; PMID: 31431832).

Comment: Insufficient discussion of cultural factors influencing KAP.
Response:
A specific subsection addressing cultural influences on sunscreen use and self-examination behaviours (e.g., cosmetic vs. preventive motivation, gender norms) has been added to the Discussion.

Comment: Expand implications for policy-making and public health in Malaysia.
Response:
We have added recommendations emphasizing targeted public health strategies that teach correct sunscreen use, encourage skin self-examination, and involve universities, primary care, and social media campaigns tailored to Malaysian cultural contexts.

Comment: Exclusion of Healthcare Professionals restricts interpretation; discuss impact and relevance of healthcare education.
Response:
We agree and have expanded the Limitations to explain that excluding healthcare workers prevents comparison with clinically trained populations. We also highlight that even healthcare trainees show inadequate practices (e.g., 61.5% never performed self-examination in Jordan; PMID: 35198247), supported by regional findings (PMID: 31431832). The need to assess KAP among healthcare professionals is now emphasized.

Comment: More references are needed in Discussion.
Response:
Additional studies have been cited to strengthen the discussion, particularly in sections addressing cultural behaviour, preventive practices, and regional comparisons.

Comment: Sample predominantly young, urban, and Chinese limits representativeness.
Response:
These points have now been explicitly addressed in the Limitations section, noting potential overrepresentation from online sampling and ethnicity imbalance. We also recommend stratified sampling and rural outreach in future research.

Response to Reviewer 3

Comment 1: Objectives need to state clear, quantifiable goals.
Response:
Thank you for the constructive recommendation. The study aims have been revised to clearly specify quantifiable objectives, including examining differences in KAP across demographic groups and identifying factors associated with preventive practices. These revisions now appear in the Introduction and Abstract.

Comment 2: Social media snowball sampling limits generalisability; recommend stratified sampling.
Response:
We agree with this important point. The Discussion and Limitations sections have been revised to explicitly state the potential overrepresentation of urban, young, and more educated participants due to online snowball sampling. We have also recommended probability-based or stratified sampling techniques for future Malaysian studies to improve representativeness.

Comment 3: Validation specifics of KAP-SC-Q should be summarised.
Response:
A summary table of key psychometric properties (including Cronbach's α and factor structure indices) has now been added as supplementary material to ensure transparency.

Comment : Report precise p-values, confidence intervals, and logistic regression.
Response:
Confidence intervals and precise p-values are now consistently presented in the Abstract and Results. Regarding logistic regression, thank you for this insightful suggestion. Logistic regression is typically applied when the primary objective includes identifying predictors of an outcome. Our study, however, was designed as a descriptive cross-sectional assessment to determine the levels of knowledge, attitudes, and practices (KAP) toward skin cancer prevention in the general Malaysian population, without modelling predictors. The statistical tests used (independent t-tests and chi-square analyses) were therefore consistent with the study aims and ethical protocol.
Expanding into multivariable modelling at this stage would introduce analyses beyond the scope of the approved study design and could risk overinterpretation of the data. For these reasons, logistic regression was not added, and our results remain focused on describing KAP distributions across demographic groups using appropriate inferential tests.

Comment: Strengthen discussion with regional comparisons and cultural behaviours.
Response:
Additional comparisons with Southeast Asian literature (including Thailand, Indonesia, and Singapore) have been added, along with expanded discussion on cultural beliefs affecting sunscreen use, tanning perceptions, and gender differences in preventive practices.

Comment: Expand limitations to include sampling bias, self-report bias, and cross-sectional design.
Response:
These points have been incorporated into the Limitations section, including online sampling biases, ethnicity imbalance, risk of social desirability bias from self-reporting, and inability to infer causality due to the cross-sectional design.

Comment : Formatting, language, and tense consistency.
Response:
The manuscript has been edited for tense consistency (methods and results written in past tense) and grammatical clarity.
